# Functional connectivity of thalamic nuclei during sensorimotor task-based fMRI at 9.4 Tesla

**DOI:** 10.3389/fnins.2025.1568222

**Published:** 2025-05-13

**Authors:** Edyta Charyasz, Michael Erb, Jonas Bause, Rahel Heule, Benjamin Bender, Vinod Kumar Jangir, Wolfgang Grodd, Klaus Scheffler

**Affiliations:** ^1^Department for High Field Magnetic Resonance, Max Planck Institute for Biological Cybernetics, Tübingen, Germany; ^2^Graduate Training Centre of Neuroscience, International Max Planck Research School, University of Tübingen, Tübingen, Germany; ^3^Department of Biomedical Magnetic Resonance, University of Tübingen, Tübingen, Germany; ^4^Center for MR Research, University Children's Hospital, Zürich, Switzerland; ^5^Department of Neuroradiology, Diagnostical, and Interventional Neuroradiology, University Hospital of Tübingen, Tübingen, Germany

**Keywords:** fMRI, thalamus, ultra-high field fMRI, high-resolution imaging, thalamic nuclei, sensorimotor, tactile, motor

## Abstract

The thalamus is the brain’s central communication hub, playing a key role in processing and relaying sensorimotor and cognitive information between the cerebral cortex and other brain regions. It consists of specific and non-specific nuclei, each with a different role. Specific thalamic nuclei relay sensory and motor information to specific cortical and subcortical regions to ensure precise communication. In contrast, non-specific thalamic nuclei are involved in general functions such as attention or consciousness through broader and less targeted connections. In the present study, we aimed to investigate the functional connectivity patterns of the thalamic nuclei identified in our previous study as being involved in motor (finger-tapping) and sensory (finger-touch) tasks. The results of this study show that thalamic nuclei are not static hubs with a predefined role in neural signal processing, as they show different task-specific functional connectivity patterns in the anterior, middle, lateral, and posterior thalamic nuclei. Instead, they are all functional hubs that can flexibly change their connections to other brain regions in response to task demands. This work has important implications for understanding task-dependent functional connectivity between thalamic nuclei and different brain regions using task-based fMRI at 9.4 Tesla.

## Introduction

The human brain is a complex and interconnected network of approximately 86 billion neurons (Azevedo et al., 2009), where coordinated activity between different brain regions enables functional processing in the brain. The cerebral cortex, for instance, is responsible for sensory processing, motor coordination, balance, motor learning and higher-level cognitive tasks such as memory, language, perception, and emotional regulation (Rolls, 2016). Similarly, the cerebellum is involved in motor coordination, balance, motor learning, cognitive processes, and emotional regulation (Manto et al., 2012; De Zeeuw and Ten Brinke, 2015; Prati et al., 2024; Rudolph et al., 2023). However, a key role in subcortical–cortical and cortico-cortical functional communication is played by the thalamus, a small structure located deep in the brain that relays sensory, cognitive and motor signals throughout the brain (Sherman and Guillery, 2006; Shine et al., 2023; Haber and McFarland, 2001; Sherman, 2007). The thalamus is composed of several distinct nuclei with specific and non-specific connections to cerebral and cerebellar areas (Jones, 1983, 1998; Zhou et al., 2011; Ward, 2013). Therefore, a detailed understanding of the functional connectivity between these nuclei and different brain regions is essential to gain insight into brain function and cognitive processes.

The functional and structural connectivity of thalamic nuclei has been investigated in numerous functional magnetic resonance imaging (fMRI) studies (Kumar et al., 2017; Mastropasqua et al., 2015; Kark et al., 2021) and DTI (Behrens et al., 2003; Jang et al., 2014; Lambert et al., 2017; Grodd et al., 2020). For example, resting-state fMRI (rs-fMRI) studies have revealed the existence of large-scale thalamocortical networks spanning many brain regions that involve multiple thalamic nuclei and their connections to different cortical regions (Beckmann et al., 2005; Zhang et al., 2008, 2010; Kumar et al., 2017, 2022). In addition to rs-fMRI studies, task-based fMRI (tb-fMRI) studies (Zhang et al., 2013; Jarrett et al., 2024; Wang, 2024; Rodriguez-Sabate et al., 2015) have played a crucial role in uncovering the functional interactions between thalamic nuclei, cortical, subcortical, and cerebellar regions during various cognitive and motor tasks. For example, Spets and Slotnick (2020) investigated the functional connectivity between anterior and mediodorsal thalamic nuclei with memory-related regions such as the prefrontal cortex, parietal cortex, visual processing regions, hippocampus, and parahippocampal cortex. Tb-fMRI studies have also elucidated the thalamocortical-cerebellar circuitry involved in motor control and coordination (Stoodley et al., 2012; Dacre et al., 2021).

Additionally, alterations in the functional connectivity of the thalamic nuclei have been observed in several neurological and psychiatric disorders, yet the causal relationship between these findings and the overarching disorders are not well understood. For example, disruptions in thalamocortical connectivity have been found to be implicated in progressive movement disorders, particularly Parkinson’s disease (Hao et al., 2020; Owens-Walton et al., 2019; Wang et al., 2021), emphasizing the importance of thalamocortical interactions in motor control. Altered thalamocortical connectivity has also been observed in cases of schizophrenia (Woodward et al., 2012; Wagner et al., 2015), depression (Sun et al., 2023; Brown et al., 2017), and attention-deficit/hyperactivity disorder (Hong, 2023; Kowalczyk et al., 2022), reflecting the impact on cognitive dysfunction.

Given the functional importance of the thalamic connectivity with different brain areas, the present study aims to investigate the functional connectivity of the thalamic nuclei during active motor (finger-tapping) and passive (tactile-finger) sensory tasks using high-resolution fMRI datasets acquired in our previous study (Charyasz et al., 2023). In this earlier study, we characterized functional localization and incidence of activation in thalamic nuclei involved in sensorimotor processes and assessed intersubject variability and reproducibility using tb-fMRI at 9.4 T. Using GLM analysis, we identified distinct activation patterns within the group of lateral nuclei (VPL, VA, VLa, and VLp) and the group of pulvinar nuclei (PuA, PuM, and PuL) during both tasks. We also observed functional activation in the intralaminar nucleus group (CM and Pf) during the finger-tapping task. Starting from these findings, we now explore the functional connectivity between the identified thalamic nuclei and various cortical and subcortical areas as well as the cerebellum in this work. This may significantly enhance understanding of the complex functional interactions of thalamic nuclei involved in sensorimotor processing.

## Materials and methods

### Participants

Our participant pool included eight healthy right-handed adults, with normal or corrected-to-normal vision, and an average age of 27 years (ranging from 21 to 34 years; five of whom were female). Ethical approval for this study was obtained from the local research ethics committee, and all participants provided written informed consent prior to participation.

### Experimental procedure

Each single session involved each participant to actively complete two fMRI block design tasks using their right hand: the tactile-finger task and the finger-tapping task. Please refer to our previous work (Charyasz et al., 2023) for a detailed description of the experimental setup and pre-processing pipeline used in this study.

### Tactile-finger task

The tactile-finger stimulation paradigm consisted of 12 cycles, with each cycle divided into alternating blocks of tactile stimulation (ON phase) and rest (OFF phase). Each block lasted a total of 40 s, equally divided into 20-s intervals for the ON and OFF phases, respectively. The tactile stimulation was delivered by air pulses through an inflatable finger clip, simultaneously applied to the thumb, index finger, middle finger, and ring finger. These air pulses, generated at a pressure of 2.5 bar, induced the displacement of the pneumatic membrane toward the skin surface for a duration of 250 milliseconds. To ensure a consistent and rhythmic pattern of stimulation across all fingers, the frequency of pulse delivery was set at 1 Hz. To prevent habituation and maintain participants’ attention, a random number of pulses (ranging from 0 to 4) was intentionally (deliberately) skipped within each stimulation block. This resulted in an average of 210–240 air pulses being delivered to each fingertip during each run. Participants were instructed to report the total number of blocks in which pulses were missing during the breaks between stimulation runs. Throughout the entire experiment, participants were lying still and keeping their eyes fixed on a black fixation cross on the screen.

### Finger-tapping task

The experimental design involved a visually-guided finger-tapping paradigm consisting of 12 visually cued cycles, each with a duration of 41 s. These cycles were divided into alternating blocks of finger tapping (ON phase) and rest (OFF phase) with each phase lasting 20 s. Each block of movement was preceded by a preparatory interval of 1 s, which was provided to ensure readiness. Participants were instructed to tap their right fingers sequentially, starting with the index finger, followed by the middle finger, ring finger, and little finger, against the thumb. The tapping rate was controlled by a visual cue in the form of a blinking arrow, with an approximate frequency of 2.5 Hz. During the designated rest blocks, participants were explicitly instructed to keep their eyes fixed on the black fixation cross, refraining from any voluntary movements.

### MR data acquisition

In a single session, both anatomical and functional images were acquired using a 9.4 T whole-body MRI scanner (Siemens Healthineers, Erlangen, Germany) with an in-house-built head-coil equipped with 16 transmit and 31 receive channels (Shajan et al., 2014). In a separate session utilizing a Siemens Healthineers Prisma Fit 3 T whole-body MRI scanner with a 64-channel head coil, high-resolution whole-brain anatomical images were acquired to facilitate the precise segmentation of the cortical and thalamic regions.

#### 9.4 T imaging

High-resolution T1-weighted images were acquired using a magnetization-prepared rapid acquisition gradient echo (MPRAGE) sequence with the following parameters: inversion repetition time (TR) = 3.8 s, echo time (TE) = 2.50 ms, flip angle (FA) = 6°, field of view (FOV) = 192 mm, 288 sagittal slices covering the entire brain, voxel size = 0.6 × 0.6 × 0.6 mm^3^, GRAPPA acceleration factor (R) = 2 × 2, and partial Fourier in slab duration = 6/8.

Task-based fMRI scans were collected using a 2D gradient-echo multi-band (MB) echo-planar imaging (GE-EPI) sequence with the following parameters: TR = 2 s; TE = 22 ms; FA = 50°; FOV = 198 mm; 86 interleaved slices per volume; voxel size = 1.25 × 1.25 × 1.25 mm^3^, *R* = 4, MB factor = 2, bandwidth = 1666 Hz/Px, and anterior–posterior phase encoding. For precise distortion correction, a set of 10 volumes with reversed phase encoding (posterior–anterior) MB-GE-EPI scans was acquired, employing the exact same parameters as during the functional scans. Each subject underwent seven runs of the tactile-finger task (255 volumes, ~8.5 min per run), and a single run of the finger-tapping task (265 volumes, ~9 min).

#### 3 T imaging

A comprehensive set of high-resolution T1-weighted and T2-weighted images were collected for each participant. The T1-weighted images were acquired using a magnetization-prepared rapid acquisition gradient echo (MPRAGE) sequence with TR = 2.4 s, TE = 2.22 ms, FA = 8°, FOV = 256 mm, and voxel size of 0.8 × 0.8 × 0.8 mm^3^. This acquisition yielded 208 sagittal slices. Additionally, the T2-weighted images were obtained using a 3D fast spin echo sequence with TR = 3.2 s, TE = 563 ms, FOV = 256 mm, and the same voxel size and slice number as the T1-weighted images.

### MRI data analysis

#### Data pre-processing

Both the task-based functional and structural images were preprocessed following the previously described methods (Charyasz et al., 2023). Briefly, the initial five volumes of the functional images were excluded, followed by correction for slice-time and head motion using the SPM12 software (R7771).^1^ Furthermore, image distortions were corrected using the TopUp (Andersson et al., 2003) tool from the FSL package (Smith et al., 2004), while NORDIC (Moeller et al., 2021) denoising was employed to correct for thermal noise fluctuations. The resulting distortion-corrected datasets were subsequently co-registered with the anatomical data and spatially smoothed with a 2.5 mm full-width-at-half-maximum Gaussian kernel.

The 3 T anatomical images, comprising T1-weighted and T2-weighted scans, were preprocessed with the FreeSurfer (version 6.1)^2^ (Fischl et al., 1999) software package (version 6.1). The recon-all function within FreeSurfer was utilized to perform a thorough whole-brain segmentation. To accurately identify thalamic nuclei, a probabilistic thalamic segmentation algorithm (Iglesias et al., 2018), integrated within FreeSurfer, was employed. Additionally, the ACAPULCO (Kerestes et al., 2022) processing pipeline was applied specifically to the T1-weighted images for cerebellar segmentation. This involved utilizing the ACAPULCO tool to segment the cerebellum into lobules, enabling a detailed sub-segmentation of the cerebellar structure. The resulting segmentation outcomes served as regions of interest (ROIs) for further analysis.

### Regions-of-interest (ROIs) selection

In accordance with the experimental design, which included tactile and motor tasks visually cued with attentional engagement, 66 anatomical ROIs (Table 1) were selected to investigate cortical-thalamic-cerebellar functional connectivity. Among these ROIs, 17 bilateral thalamic nuclei were chosen based on their prior identification (Charyasz et al., 2023) and their relevance to the research context. In addition, a set of 32 anatomical ROIs (16 in each hemisphere) comprising brain regions involved in sensorimotor, visual, and attentional signal processing were included. More specifically, 24 cortical ROIs were obtained from the Desikan-Killiany-Tourville (DKT) atlas, while the remaining 8 cerebellar ROIs were derived from the ACAPULCO segmentation.

**Table 1:** The complete list of the 33 bilateral ROIs and their abbreviations.

Thalamic nuclei	Anteroventral (AV), lateral dorsal (LD), lateral posterior (LP), ventral anterior (VA), ventral lateral anterior (VLa), ventral lateral posterior (VLp), ventral posterior lateral (VPL), centromedian (CM), parafascicular (Pf), lateral subdivision of mediodorsal thalamus (MDl), medial subdivision of mediodorsal thalamus (MDm), lateral geniculate (LGN), medial geniculate (MGN), anterior pulvinar (PuA), inferior pulvinar (PuI), later pulvinar (PuL), medial pulvinar (PuM)
Cortical regions	Paracentral gyrus (PCG), postcentral gyrus (PoCG), precentral gyrus (PrCG), superior parietal lobule (SPL), inferior parietal lobule (IPL), cuneus (Cun), pericalcarine (PCAL), lingual gyrus (LING), insula (INS)
Subcortical regions	Caudate (Cd), Putamen (Pu), Pallidum (Pd)
Cerebellum	Lobule I-II (Lob I-III), Lobule IV (Lob IV), Lobule V (Lob V), Lobule VI (Lob VI)

### Functional connectivity analysis

Task-based functional connectivity analyses were carried out using the CONN (Whitfield-Gabrieli and Nieto-Castanon, 2012) release 22a (Nieto-Castanon and Whitfield-Gabrieli, 2022) within MATLAB 2017b (The MathWorks, Inc., Natick, MA, USA). The preprocessed fMRI data were used as input to the CONN toolbox to undergo denoising and subsequent ROI-to-ROI functional connectivity analysis.

#### Denoising

Prior to connectivity analysis, the functional data were denoised to isolate intrinsic connectivity by removing nuisance signals and task-related coactivations. The CONN’s default denoising pipeline (Nieto-Castanon, 2020a) consists of two main steps: linear regression of confounding effects and temporal band-pass filtering. In the regression step, physiological noise correction was achieved by regressing out five CompCor noise components from the white matter time series and five CompCor noise components from the CSF time series. In addition, 12 motion regressors and their first-order derivatives were included to account for any motion-related confounds. Session and task effects (motor and tactile) were modeled as additional nuisance regressors to separate task-related coactivations from genuine functional connectivity patterns. This included constant and linear trends within each session to account for low-frequency drifts. Task-related effects were defined by boxcar functions convolved with a canonical haemodynamic response function (HRF) and their temporal derivatives. This approach aimed to minimize the influence of consistent task-related activations, initial magnetization transients, and slow signal fluctuations that might otherwise bias connectivity estimates. Following regression, linear detrending and band-pass filtering (0.008 Hz and 0.09 Hz) were applied on the BOLD timeseries (Hallquist et al., 2013) to reduce low- and high-frequency noise. CompCor (Behzadi et al., 2007; Chai et al., 2012) noise components within white matter and CSF were estimated by computing the average BOLD signal as well as the largest principal components orthogonal to the BOLD average within each subject’s eroded segmentation masks. From the number of noise terms included in this denoising strategy, the effective degrees of freedom of the BOLD signal after denoising were estimated to range from 652.7 to 667.5 (average 665.6) across all subjects (Nieto-Castanon, 2020a).

#### First-level functional connectivity analysis

Following denoising, first-level functional connectivity was calculated using the weighted ROI-to-ROI connectivity (wRRC) approach, which incorporates a weighted general linear model (wGLM). This method evaluates task condition-specific functional connectivity among a predefined set of ROIs. The wRRC approach was applied in several task-based fMRI studies (Agren and Hoppe, 2024; Doganci et al., 2023; Pereira et al., 2024).

For each subject, average BOLD time series were extracted from 66 anatomically defined ROIs (Table 1). These time series were obtained by averaging the signal across all voxels within each ROI, using pre-processed but unsmoothed functional data to preserve spatial specificity. Connectivity between each pair of ROIs was then estimated using a Weighted Least Squares (WLS) linear model. Task condition-specific temporal weights were applied to restrict the analysis to the relevant task-condition periods, including tactile stimulation, motor tasks, and their respective baselines. These weights were created by convolving a boxcar function representing each condition with an HRF.

The resulting 66 × 66 wRRC matrices were computed independently for each subject and condition. Each matrix entry represents the Fisher-transformed bivariate correlation coefficient (Z-score) between a pair of ROIs during that specific task or baseline block, reflecting the strength of their functional interaction (Nieto-Castanon, 2020b).

#### Second-level functional connectivity analysis

Second-level analyses were conducted using a GLM approach (Nieto-Castanon, 2020c). Functional connectivity estimates from the first-level wRRC analysis were used as the dependent variables. These measures reflected Fisher-transformed correlation coefficients for each pair of ROIs, computed separately for each condition (tactile stimulation, motor tasks, and their respective baselines). For each of the four conditions, a separate second-level analysis was performed using a one-sample *t*-test to examine whether the mean connectivity for each pair of ROIs significantly differed from zero across subjects. Condition-specific connectivity matrices were analysed independently to identify the brain’s functional connectivity pattern during motor and tactile task conditions independently. This approach was selected to reduce the number of statistical comparisons and to account for the limited number of subjects (*n* = 8), which limited the statistical power of within-subject contrast models.

Connection-level hypotheses were tested using multivariate parametric statistics with random effects modeling across participants and covariance estimation across measures. Statistical inference was performed at the level of individual functional connections, and results were thresholded using a family-corrected p-FDR < 0.05 connection-level threshold (Benjamini et al., 2001).

Further second-level analyses were conducted to compare each task condition with its corresponding baseline (tactile stimulation > tactile baseline, motor task > motor baseline). The same FDR-corrected threshold of *p* < 0.05 used in the condition-specific analyses was applied. As no functional connections showed statistically significant effects, an exploratory analysis was subsequently performed using a connection level threshold of uncorrected *p* < 0.01.

#### Effect size estimation

To estimate the size of connectivity differences in response to the tactile and motor tasks, within-subject effect sizes were calculated using Cohen’s d based on the first-level wRRC matrices. For each subject, the connectivity values obtained during the baseline conditions were subtracted from the respective task conditions (tactile - tactile baseline; motor - motor baseline), resulting in a subject-level difference matrix for each task. Group-level means, and standard deviations of these differences were then computed across subjects for each ROI-to-ROI connection. Cohen’s d was calculated as the ratio of the mean difference to its standard deviation, resulting in a 66 × 66 matrix of standardized effect sizes for the motor and tactile task, respectively. These matrices were used to determine the strength and direction of task-related functional connectivity changes, independent of statistical significance. Effect sizes were interpreted as per standard conventions (Cohen, 1988). In the full 66 × 66 matrices, raw Cohen’s d values were visualized without binning or thresholding, allowing a continuous representation of effect sizes across all ROI connections. For targeted thalamic visualizations, effect sizes were plotted separately for each thalamic group (anterior, medial, lateral and posterior nuclei), while excluding self-connections between thalamic ROIs. In these subgroup plots, values were categorized as follows:

- Weak effects (−0.2 < *d* < 0.2) were masked and not displayed.
- Small effects: 0.2 ≤ *d* < 0.5 or −0.5 < *d* ≤ −0.2
- Moderate effects: 0.5 ≤ *d* < 0.8 or −0.8 < *d* ≤ −0.5
- Large effects: *d* ≥ 0.8 or *d* ≤ −0.8

These categories were visualized using a color scheme ranging from light to dark red (positive effects) and light to dark blue (negative effects) to support a clearer interpretation of the connectivity patterns within each thalamic subdivision.

## Results

The results of our ROI-based functional connectivity analysis clearly showed task-dependent changes in inter-regional functional connectivity between the selected ROIs. These changes were dependent on whether participants performed a finger-tapping task or a tactile task. First, the results of the second-level ROI-to-ROI functional connectivity analyses are presented for each task, highlighting task-specific patterns of connectivity across brain regions. The corresponding effect sizes (Cohen’s d) are then reported, providing a standardized quantification of connectivity changes relative to baseline for both motor and tactile tasks.

### Task-dependent connectivity analysis

**Figure 1:**
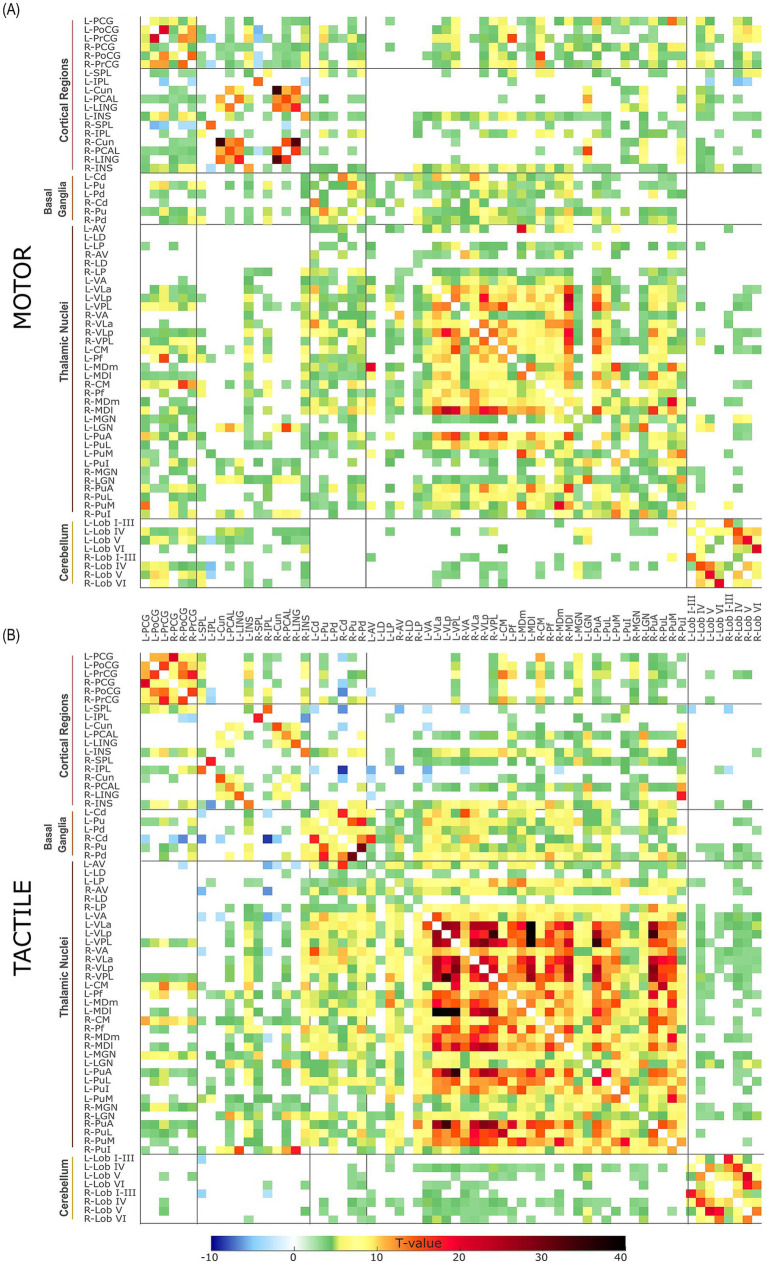
Group-level ROI-to-ROI functional connectivity matrices for the **(A)** motor and **(B)** tactile tasks across 66 regions of interest (ROIs), thresholded at p-FDR < 0.05 (connection level). ROIs are organized and labelled according to anatomical divisions (cortical regions, basal ganglia, thalamic nuclei, cerebellum), with consistent ordering along both axes. The colour scale represents *T*-values, ranging from negative (blue) to positive (black), indicating the strength of functional connectivity differences.

To capture the overall connectivity patterns, the connectivity matrices showing the task-modulated interactions between all 66 ROIs across all subjects are presented in Figure 1, providing a comprehensive representation of the connections. In general, robust connections were observed between and among thalamic, cortical, subcortical and cerebellar regions in both hemispheres. To improve clarity and organization, the results were also divided into smaller groups allowing for cross-comparison of task-specific changes within the examined brain regions.

Although the results presented and discussed below illustrate group-level connectivity patterns, subject-level ROI-to-ROI connectivity matrices obtained from the first-level analysis are provided in the Supplementary Figures S1, S2. These are included to demonstrate that task-related functional connectivity patterns were generally consistent across individual participants. However, a detailed analysis of single-subject variability, as well as task-dependent connectivity between cortical, subcortical, and cerebellar regions at the group level, is not pursued further here, as both are beyond the scope of the present study.

As mentioned in the Methods section, direct contrasts between task conditions (tactile > tactile baseline vs. motor > motor baseline) did not yield statistically significant effects at the FDR-corrected threshold (*p* < 0.05). Given this limitation, additional results obtained at an uncorrected threshold of *p* < 0.01 are presented in the Supplementary Figures S3, S4 and briefly discussed in the “Discussion” section as exploratory to orient future research.

#### Task-dependent functional connectivity of the anterior thalamic nuclei

**Figure 2:**
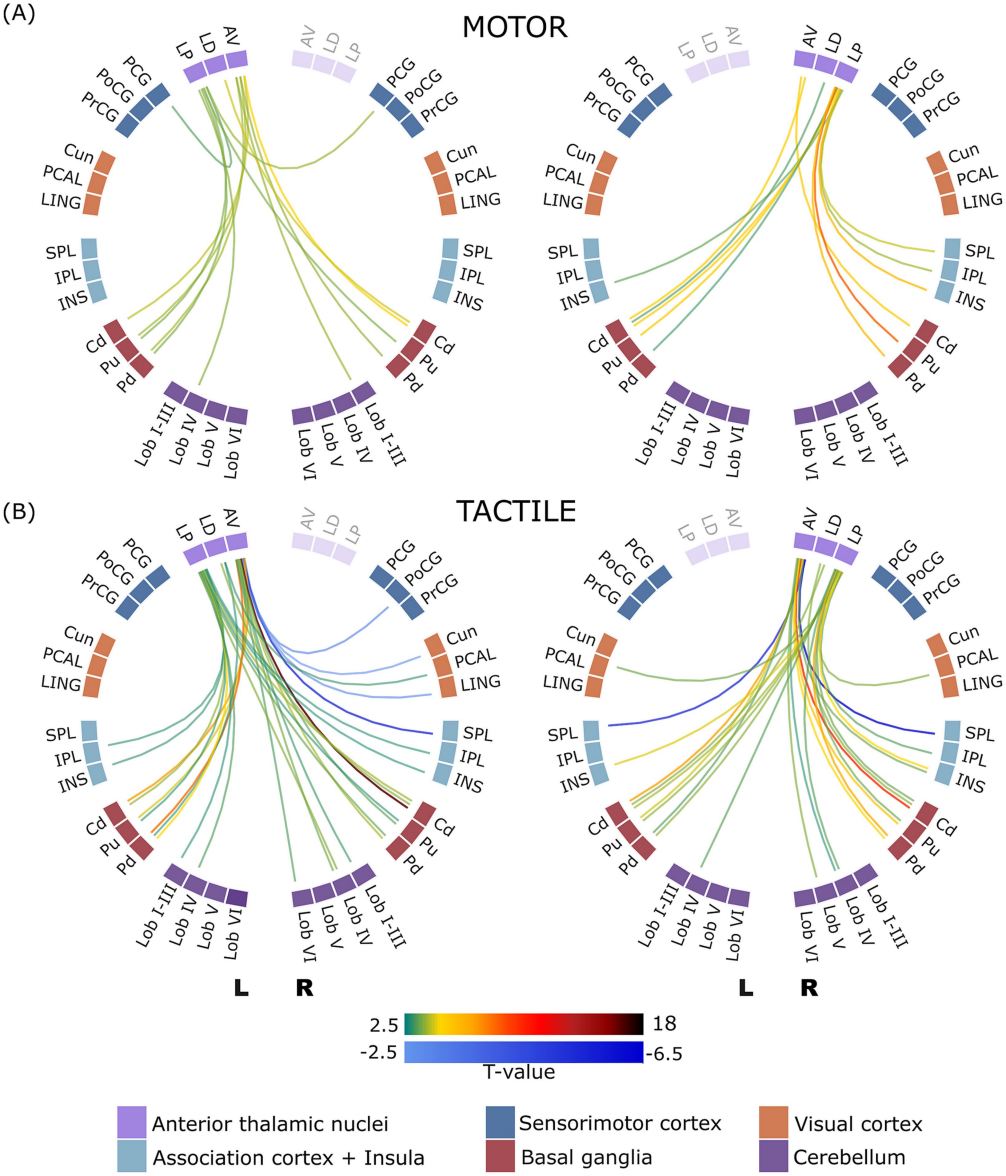
ROI-to-ROI connectome ring of functional connectivity between the anterior thalamic nuclei and other brain regions during motor **(A)** and tactile **(B)** tasks. The left panel displays connectivity patterns involving the left anterior thalamic nuclei, and the right panel shows those involving the right anterior thalamic nuclei. Only statistically significant connections are presented (corrected p-FDR < 0.05). Green to dark red lines indicate significant positive correlations, while blue lines indicate significant negative correlations. Regions of interest (ROIs) are arranged circularly and grouped by hemisphere, with clear separation between left (L) and right (R) hemisphere structures. Displayed connections are limited to those between thalamic nuclei and non-thalamic regions; intra-thalamic connectivity is not shown.

In both motor and tactile tasks, the connectivity analysis revealed increased functional connectivity between the anterior thalamic nuclei (AV, LD, and LP) and various brain regions, including cerebellar lobules and basal ganglia structures (caudate, putamen and pallidum). In the motor task, increased connectivity was observed between the right LP nucleus and the cortical regions of the bilateral insula, right SPL, and right IPL, while the left LP nucleus exhibited increased connectivity with the bilateral PCG. In the tactile task, increased connectivity was observed between the bilateral LP nuclei and the bilateral insula. In addition, decreased connectivity was found between the left AV nucleus and right cortical regions including the cuneus, lingual gyrus, SPL and PoCG, and between the right AV nucleus and bilateral SPL. In general, the tactile task exhibited a higher number of significant connections compared to the motor task. The comprehensive details of these observed connections, including the specific brain regions involved and the corresponding t-values, are summarized in Supplementary Table S1. In addition, the graphical representation of these connectivity patterns is shown in Figure 2.

#### Task-dependent functional connectivity of the medial thalamic nuclei

**Figure 3:**
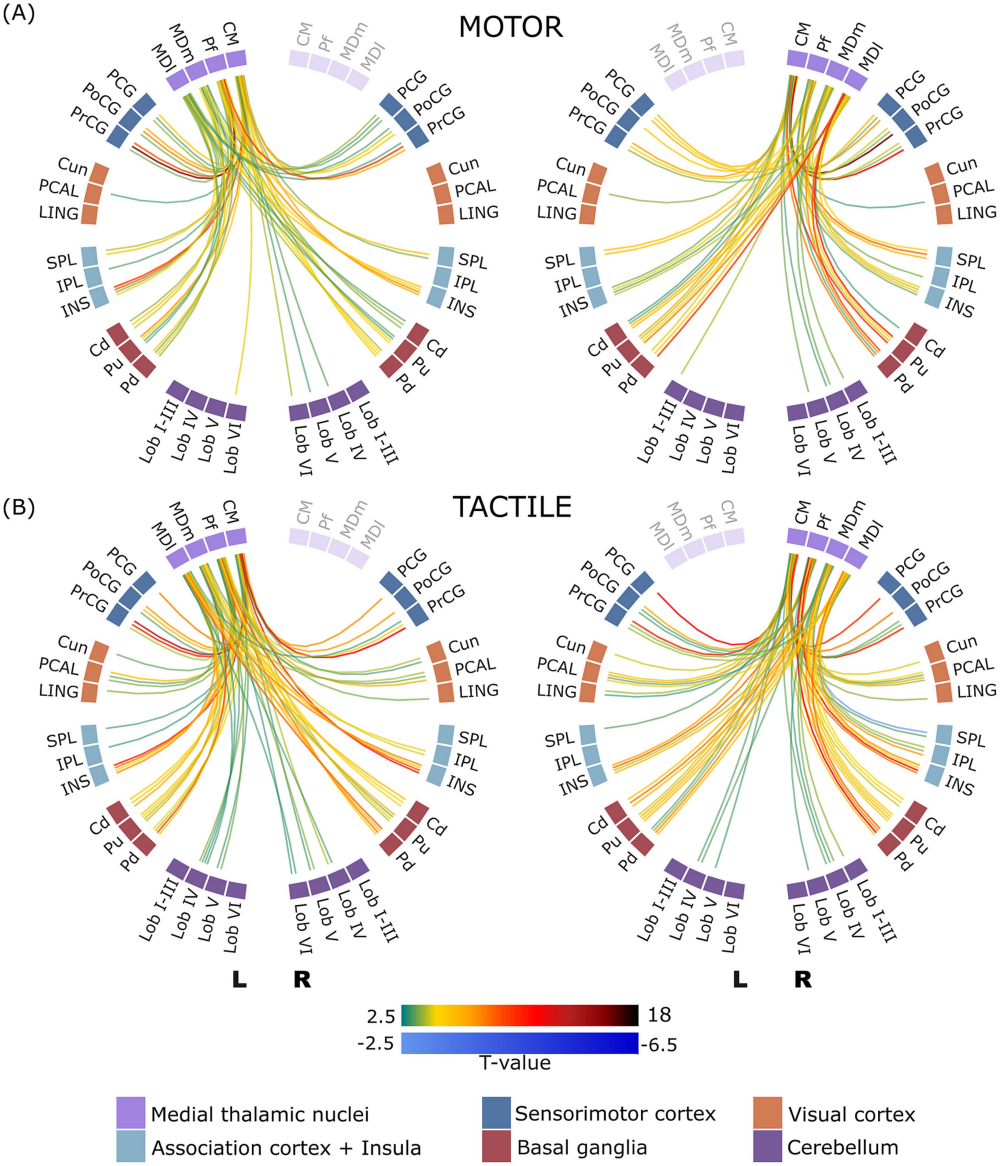
ROI-to-ROI connectome ring of functional connectivity between the medial thalamic nuclei and other brain regions during motor **(A)** and tactile **(B)** tasks. The left panel displays connectivity patterns involving the left medial thalamic nuclei, and the right panel shows those involving the right medial thalamic nuclei. Only statistically significant connections are presented (corrected p-FDR < 0.05). Green to dark red lines indicate significant positive correlations, while blue lines indicate significant negative correlations. Regions of interest (ROIs) are arranged circularly and grouped by hemisphere, with clear separation between left (L) and right (R) hemisphere structures. Displayed connections are limited to those between thalamic nuclei and non-thalamic regions; intra-thalamic connectivity is not shown.

Analysis of the connectivity between the medial thalamic nuclei (CM, Pf, MDm and MDl) and other brain regions revealed distinct variations in the connectivity patterns between the motor and tactile tasks are shown in Figure 3. The detailed Supplementary Table S2 provides a comprehensive overview of all significant connections and their corresponding statistical values.

Increased connectivity between cerebellar lobules and thalamic nuclei was observed in both motor and tactile tasks, with notable differences in the specific nuclei involved. In the motor task, connectivity was predominantly observed with the CM and MDm nuclei, whereas in the tactile task all four nuclei of the medial thalamic group showed increased connectivity. The number of significant connections was notably lower in the motor task than in the tactile task. In both tasks, there was a significant increase in functional connectivity between all four nuclei of the medial group and bilateral basal ganglia structures, including the putamen, pallidum and caudate, with a similar number of connections.

The medial group nuclei connectivity patterns differed between the two tasks. Specifically, connectivity between two nuclei (CM and PF) and both bilateral cunei and bilateral lingual areas was observed in the tactile task, whereas no such connectivity was observed in the motor task. There were also significant differences in connectivity to the PCAL region. In the tactile task, increased connectivity was observed between all four nuclei and bilateral PCAL. In contrast, only two connections were found in the motor task, specifically between the bilateral CM nuclei and the left PCAL.

Interestingly, the most pronounced differences in connectivity patterns were found in the sensorimotor cortical regions, including the PCG, PoCG and PrCG. Both tasks resulted in connections between bilateral PCG and bilateral CM, albeit a greater number of connections were observed in the motor task. The bilateral MDL and MDm, however, only resulted in statistically significant connections during the motor task (not the tactile task). In the motor task, the bilateral PoCG showed significant connections with all four medial thalamic nuclei, whereas the bilateral PrCG showed significant connections with three thalamic nuclei (CM, Pf and MDl). Similarly, during the tactile task, the bilateral PoCG and PrCG exhibited significant connections with all four nuclei.

#### Task-dependent functional connectivity of the lateral thalamic nuclei

**Figure 4:**
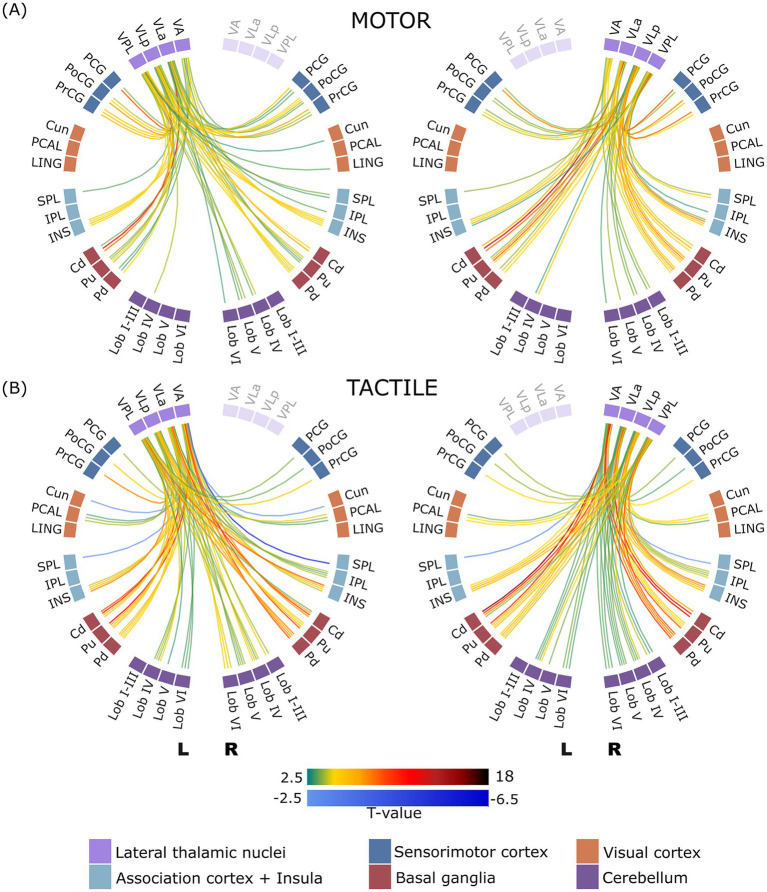
ROI-to-ROI connectome ring of functional connectivity between the lateral thalamic nuclei and other brain regions during motor **(A)** and tactile **(B)** tasks. The left panel displays connectivity patterns involving the left lateral thalamic nuclei, and the right panel shows those involving the right lateral thalamic nuclei. Only statistically significant connections are presented (corrected p-FDR < 0.05). Green to dark red lines indicate significant positive correlations, while blue lines indicate significant negative correlations. Regions of interest (ROIs) are arranged circularly and grouped by hemisphere, with clear separation between left (L) and right (R) hemisphere structures. Displayed connections are limited to those between thalamic nuclei and non-thalamic regions; intra-thalamic connectivity is not shown.

Examining the connectivity between the lateral thalamic nuclei (VA, VLa, VLp and VPL) and other brain regions revealed interesting variations in the connectivity patterns across the motor and tactile tasks, as shown in Figure 4. In this section, we provide a comprehensive overview of the observed connectivity patterns in each task, together with the corresponding statistical values, as summarized in Supplementary Table S3.

Consistent with the groups of anterior and medial thalamic nuclei, the motor and sensory tasks showed similar patterns of increased connectivity between the cerebellar lobes and the lateral thalamic nuclei. Notably, with fewer significant connections in the motor task, the strength of the connections was similar in both tasks. Both motor and tactile tasks showed increased connectivity between subcortical regions (bilateral putamen, caudate and pallidum) and all four bilateral nuclei (VA, VLa, VLp, and VPL). However, compared to the motor task, the tactile task exhibited significantly higher connectivity strength. The bilateral insula showed increased connectivity with the bilateral nuclei of the lateral nuclear group in both motor and tactile tasks. The strength of these connections, however, was comparable between the tasks.

Increased connectivity between the bilateral PCAL and the bilateral VLa, VLP and VPL nuclei was only found during the tactile task, whereas the increased connectivity between the left VPL nucleus and the right lingual gyrus was only observed during the motor task. In the tactile task, the right IPL showed increased connectivity with all four nuclei bilaterally, whereas in the motor task connectivity was limited to a single connection with the left VLa nucleus. On the other hand, connectivity between the left VPL nucleus and the right cuneus increased during the motor task. In addition, several negative connections appeared only during the tactile task. These included a reduction in connectivity between the left and right SPL and the bilateral VA nucleus, and between the bilateral cuneus and the left VA nucleus.

Similar to the medial group of thalamic nuclei, the most significant differences in connectivity patterns between tasks were found between the lateral group of thalamic nuclei and sensorimotor cortical regions (PCG, PoCG and PrCG). Connectivity analysis revealed differences between the motor and tactile tasks in the number of pairwise connections. A total of 33 and 14 pairwise connections were observed in the motor task and the tactile task, respectively. In the motor task, increased connectivity was observed between the bilateral PCG and the VLa, VLp and VPL nuclei, with the strongest connections observed with the bilateral VPL. The PoCG reflected increased connectivity between the bilateral and all four nuclei. PrCG reflected enhanced connectivity bilaterally with the VLa, VLp, and VPL nuclei. Alternatively, the tactile task had different connectivity patterns. The tactile task reflected increased connectivity between the PCG, PrCG, and PoCG bilaterally with the VPL nucleus. The left PrCG also showed increased connectivity with the left VA and left VLp nuclei in the tactile task. Overall, the motor task showed a higher number and strength of connections with thalamic nuclei, particularly involving Vla, VLp, and VPL, compared to the tactile task, which involved mainly the VPL nucleus with fewer connections.

#### Task-dependent functional connectivity of the posterior thalamic nuclei

Consistent with findings in other thalamic nuclei groups, analysis of the posterior group (MGN, LGN, PuA, PUL, PuM, and PuI) revealed a significantly lower number of pairwise connections with subcortical and cerebellar regions in the motor task compared to the tactile task. Increased connectivity between the cerebellar lobules and the thalamic nuclei was observed in both the motor task and the tactile task, with notable differences in the specific nuclei involved. Enhancement of connectivity between the posterior group of nuclei (left LGN, bilateral MGN, bilateral PuA and PuM) and bilateral cerebellar lobules (I-III, IV and V) was observed in the motor task, whereas connectivity between bilateral nuclei (LGN, MGN, PuA, PuL and PuM) and bilateral lobules IV-VI was found in the tactile task. In both tasks, a significant increase in functional connectivity was observed between all nuclei of the posterior group and the bilateral nuclei of the basal ganglia, including the putamen, pallidum and caudate, with similar strength of connections.

Increased connectivity of thalamic nuclei with specific cortical regions was found in both tasks. Connectivity analysis of the tactile task revealed a greater strength and number of connections in the bilateral insula, whereas the bilateral SPL showed a greater number of connections in the motor task. The bilateral PCAL showed a greater strength of connectivity in the motor task but, interestingly, with a greater number of connections in the tactile task. The cuneus and the lingual gyrus showed consistent connectivity patterns bilaterally in both tasks. Notably, the bilateral IPL only showed increased connectivity in the tactile task.

Similar to the previous thalamic nuclei groups, differences between tasks in the pattern of connectivity with sensorimotor cortical regions were revealed. An increase in connectivity between the distinct nuclei of the lateral group and the bilateral PCG, PoCG and PrCG was observed in both tasks. However, the number of pairwise connections was higher in the motor task (46 connections) than in the tactile task (37 connections). In the motor task, the bilateral PCG showed stronger connections with all nuclei belonging to the lateral group. However, in the tactile task, no connectivity was observed between the PCG and LGN and PuM nuclei. Both tasks showed comparable connectivity strength between the PoCG and other nuclei, with no connections to LGN nuclei in either task. Similarly, the bilateral PrCG showed no connectivity with PuM nuclei in either task, and with no PuI in the tactile task.

**Figure 5:**
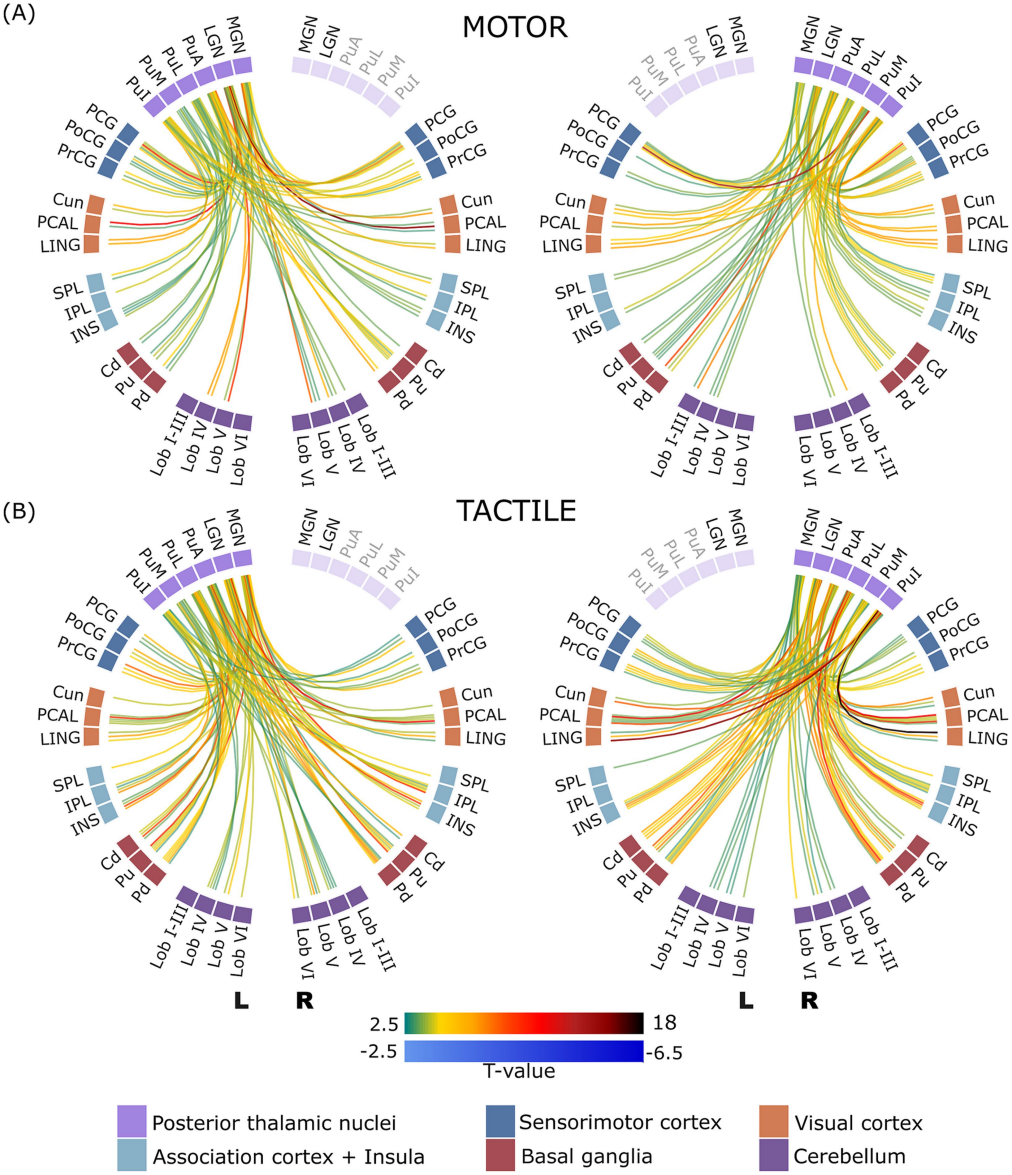
ROI-to-ROI connectome ring of functional connectivity between the posterior thalamic nuclei and other brain regions during motor **(A)** and tactile **(B)** tasks. The left panel displays connectivity patterns involving the left posterior thalamic nuclei, and the right panel shows those involving the right posterior thalamic nuclei. Only statistically significant connections are presented (corrected p-FDR < 0.05). Green to dark red lines indicate significant positive correlations, while blue lines indicate significant negative correlations. Regions of interest (ROIs) are arranged circularly and grouped by hemisphere, with clear separation between left (L) and right (R) hemisphere structures. Displayed connections are limited to those between thalamic nuclei and non-thalamic regions; intra-thalamic connectivity is not shown.

Supplementary Table S4, which provides detailed information on all detected pairwise connections, shows these task-dependent differences in connectivity. Furthermore, Figure 5 shows them visually, where Figure 5A shows the motor task and Figure 5B shows the tactile task.

### Whole-brain effect size distributions

**Figure 6:**
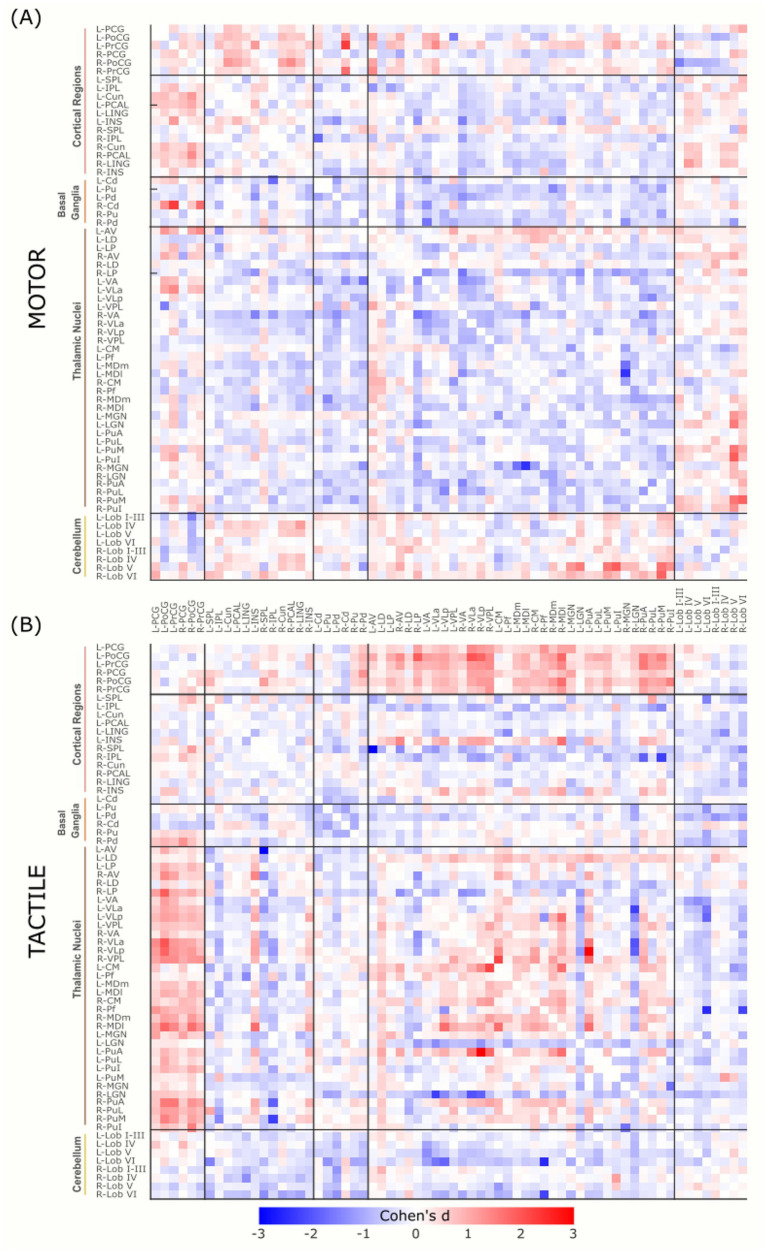
Cohen’s d effect size matrices representing task-related functional connectivity changes. Connectivity during the motor **(A)** and tactile **(B)** tasks is compared to their respective baseline conditions. Each matrix cell reflects the effect size of the difference in connectivity between pairs of ROIs. Warm colours (red) indicate increased connectivity during task relative to baseline, while cool colours (blue) reflect decreased connectivity.

Whole-brain task-related changes in functional connectivity were quantified using Cohen’s d, calculated for all ROI-to-ROI connections by comparing each task condition to its respective baseline. Effect sizes (Cohen’s d) for motor and tactile conditions (relative to their respective baselines) are shown in Figures 6A,B, respectively. The motor condition exhibited a negatively skewed distribution of connectivity changes (mean *d* = −0.12, SD = 0.45, range: −2.39 to 2.28), indicating that functional connectivity was more frequently reduced relative to baseline. A total of 940 connections exceeded the positive small-effect threshold (*d* ≥ 0.2), including 264 moderate (0.5 ≤ *d* < 0.8) and 94 large (*d* ≥ 0.8) effects. In contrast, 1,842 connections showed negative changes below *d* ≤ −0.2, including 560 moderate and 224 large negative effects, reflecting a widespread reduction in functional coupling during motor execution.

The tactile task produced a broader and more symmetrical distribution of effect sizes (mean *d* = −0.03, SD = 0.53, range: −3.03 to 3.10), with more balanced increases and decreases in connectivity. A total of 1,244 connections exceeded *d* ≥ 0.2, comprising 352 moderate and 264 large effects, suggesting substantial increases in functional connectivity compared to baseline. Negative effects were also present but less dominant than in the motor condition, with 1,586 connections below *d* ≤ −0.2, including 398 moderate and 232 large decreases. These results suggest that the motor task predominantly led to reductions in connectivity compared to baseline, whereas the tactile task evoked more balanced and widespread increases-especially for large effects-indicating broader network involvement during tactile processing.

**Figure 7:**
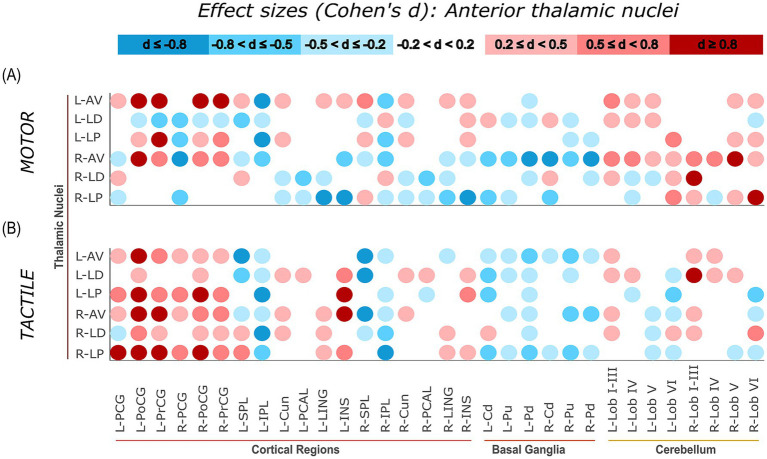
Effect sizes (Cohen’s d) representing task-related changes in functional connectivity between anterior thalamic nuclei and cortical, subcortical, and cerebellar regions. Panels **(A,B)** show the motor and tactile tasks, respectively, each compared to their corresponding baseline condition. Each circle represents the effect size of a connection between a specific anterior thalamic nucleus and a specific ROI (columns). The colour intensity indicates the direction and magnitude of the effect sizes: red shades denote increased connectivity during the task relative to baseline, while blue shades indicate decreased connectivity.

**Figure 8:**
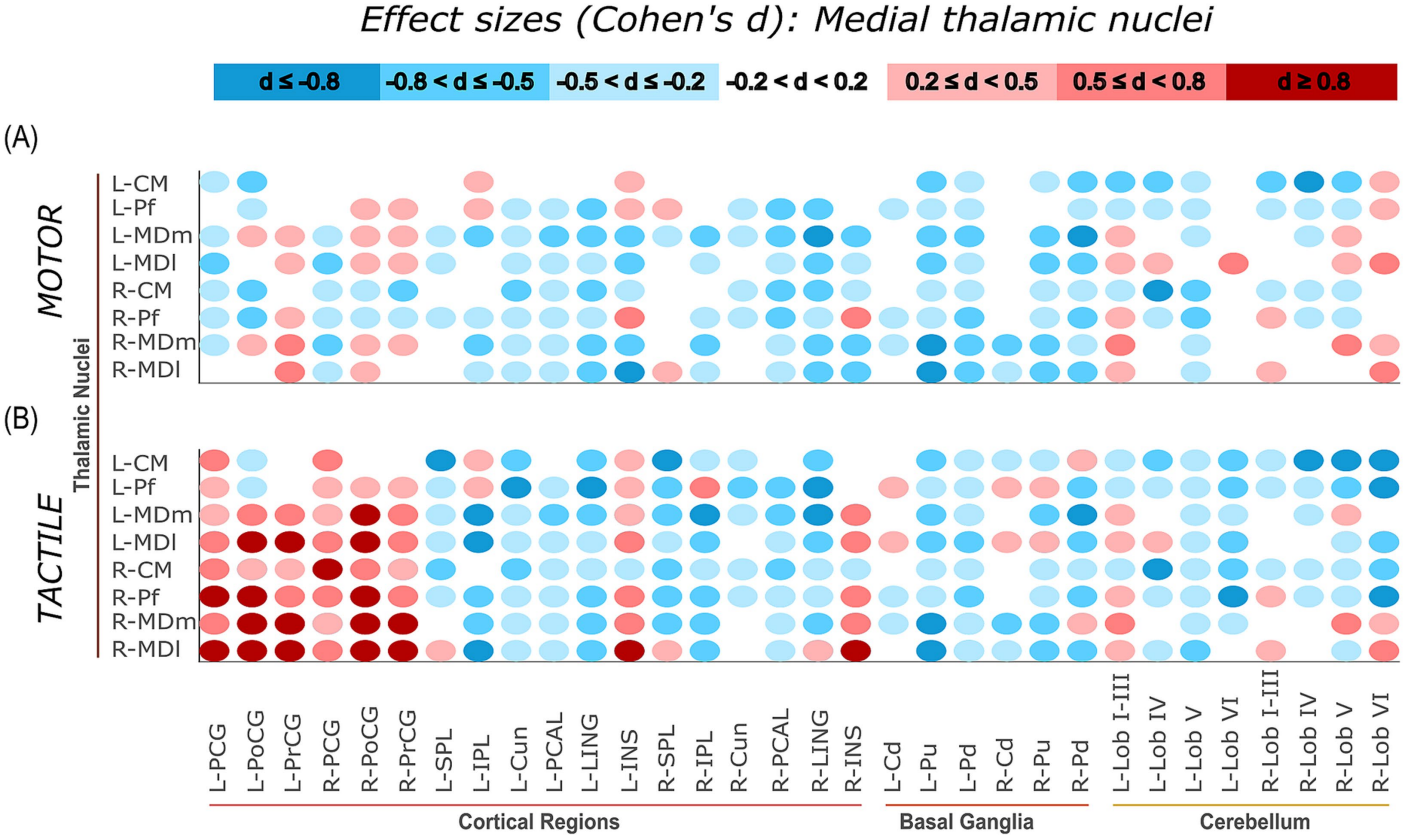
Effect sizes (Cohen’s d) representing task-related changes in functional connectivity between medial thalamic nuclei and cortical, subcortical, and cerebellar regions. Panels **(A,B)** show the motor and tactile tasks, respectively, each compared to their corresponding baseline condition. Each circle represents the effect size of a connection between a specific medial thalamic nucleus and a specific ROI (columns). The colour intensity indicates the direction and magnitude of the effect sizes: red shades denote increased connectivity during the task relative to baseline, while blue shades indicate decreased connectivity.

**Figure 9:**
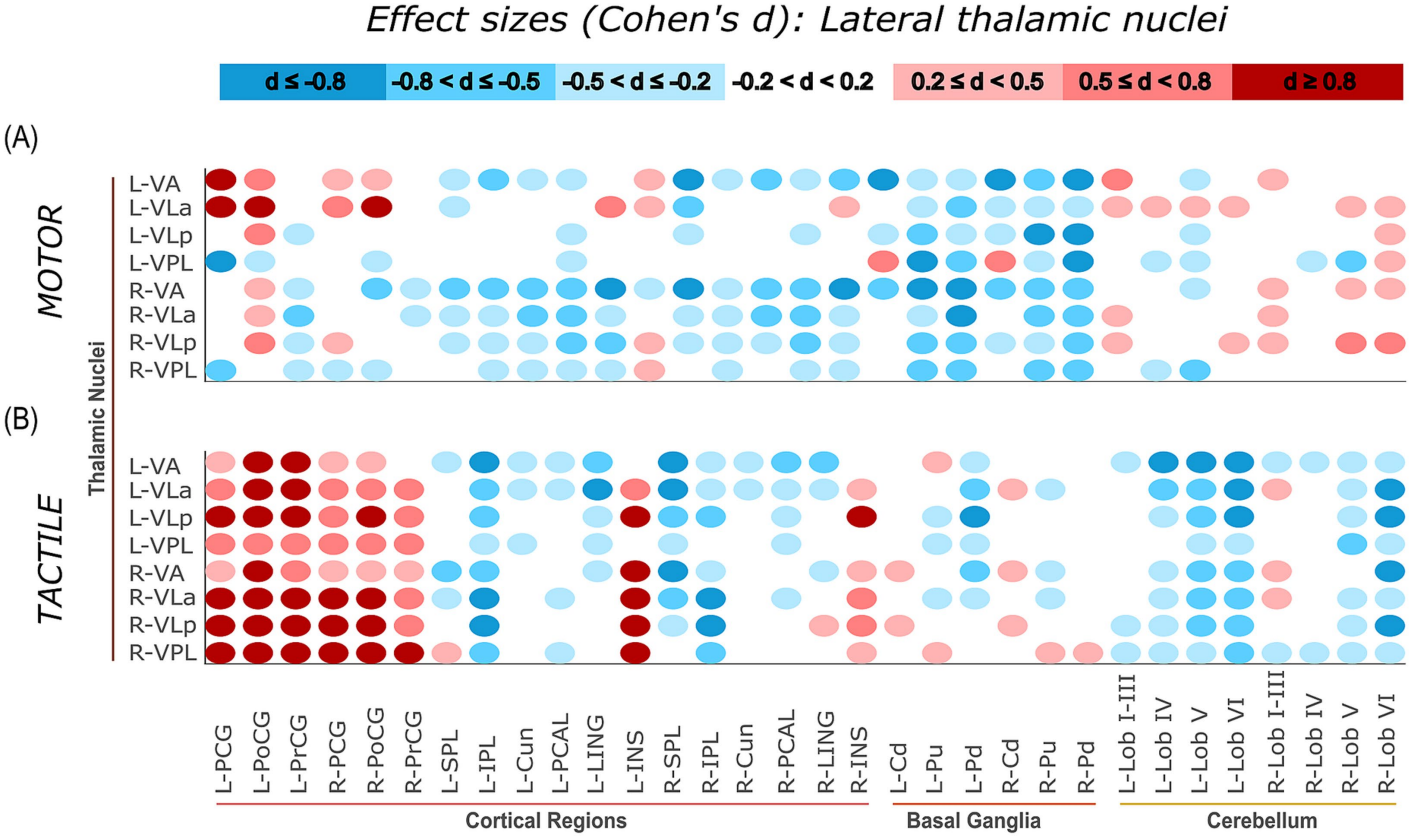
Effect sizes (Cohen’s d) representing task-related changes in functional connectivity between lateral thalamic nuclei and cortical, subcortical, and cerebellar regions. Panels **(A,B)** show the motor and tactile tasks, respectively, each compared to their corresponding baseline condition. Each circle represents the effect size of a connection between a specific lateral thalamic nucleus and a specific ROI (columns). The colour intensity indicates the direction and magnitude of the effect sizes: red shades denote increased connectivity during the task relative to baseline, while blue shades indicate decreased connectivity.

**Figure 10:**
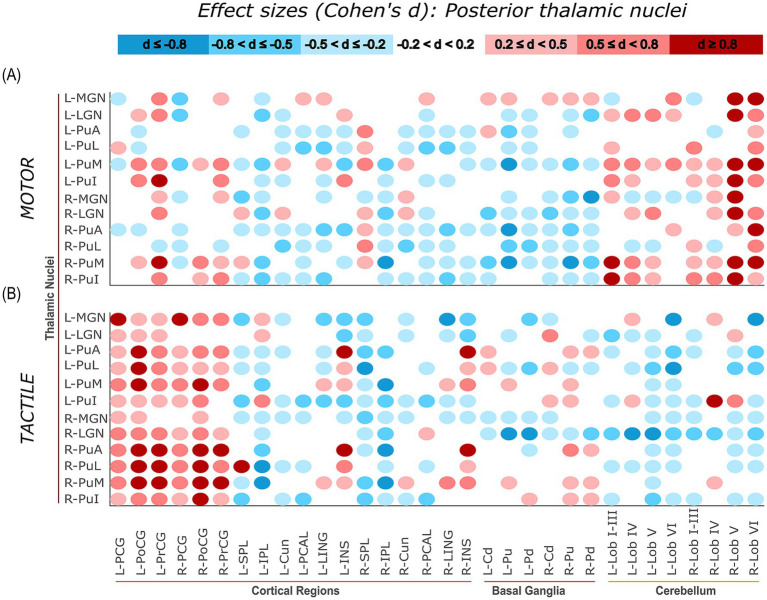
Effect sizes (Cohen’s d) representing task-related changes in functional connectivity between posterior thalamic nuclei and cortical, subcortical, and cerebellar regions. Panels **(A,B)** show the motor and tactile tasks, respectively, each compared to their corresponding baseline condition. Each circle represents the effect size of a connection between a specific posterior thalamic nucleus and a specific ROI (columns). The colour intensity indicates the direction and magnitude of the effect sizes: red shades denote increased connectivity during the task relative to baseline, while blue shades indicate decreased connectivity.

Task-related connectivity patterns varied across the four thalamic nuclei groups, with distinct shifts in effect size distributions observed between the motor and tactile conditions. The most pronounced contrasts involved connections with sensorimotor and cerebellar regions, where each thalamic group exhibited condition-specific patterns of increases and decreases in connectivity, as measured by Cohen’s *d* relative to baseline. For additional visualization, task-related effect sizes (Cohen’s d) were plotted separately for each thalamic group. Weak effects (−0.2 < *d* < 0.2) were masked, and the remaining values were grouped into small, moderate, and large effect size. This approach allows for more distinct illustration of condition-specific connectivity patterns of specific thalamic nuclei groups with cortical, subcortical, and cerebellar regions (Figures 7–10).

#### Anterior thalamic nuclei

During the tactile task, the anterior thalamic nuclei (AV, LD, LP) exhibited large task-related increases in connectivity with left sensorimotor regions. The strongest effects were observed for right LP – left PoCG (*d* = 2.05), right AV – left PoCG (*d* = 1.46), and right LP – left PCG (*d* = 1.18), indicating substantial increases in functional connectivity relative to baseline. In contrast, cerebellar connectivity was generally weaker. The strongest cerebellar effect was seen for left LD – right Lob I–III (*d* = 0.97), while others showed smaller increases or reductions, such as left LP – left Lob VI (*d* = −0.60). In the motor task, the connectivity profile shifted. Anterior nuclei showed stronger increases in connectivity with cerebellar regions, including right AV – right Lob V (*d* = 1.07), right LD – right Lob I–III (*d* = 0.96), and right LP – right Lob VI (*d* = 0.95). Connectivity with sensorimotor cortex was more variable: left AV – right PrCG showed a strong increase (*d* = 1.48), while right AV – right PCG exhibited a task-related decrease (*d* = −1.08). A visual summary of these effects is provided in Figure 7.

#### Medial thalamic nuclei

During the tactile task, the medial thalamic nuclei (CM, Pf, MDm, MDl) exhibited strong task-related increases in connectivity with bilateral sensorimotor regions, relative to baseline. The most prominent effects included right MDl – left PoCG (d = 1.92, large), right MDl – right PoCG (*d* = 1.82, large), and right MDl - left PrCG (*d* = 1.53, large), indicating task-related increases in connectivity. Cerebellar connectivity during the tactile task was predominantly negative, with effects such as right Pf – left Lob VI (*d* = −2.70, large negative), right Pf - right Lob VI (*d* = −2.38, large negative), and left CM - right Lob V (*d* = −1.29, large negative).

In the motor task, connectivity to sensorimotor regions was generally reduced or negative, including left MDl – right PCG (*d* = −0.83, moderate negative) and right Pf – left PoCG (*d* = −0.83, moderate negative). Cerebellar size effects remained low, with weak negative or small positive values. The strongest cerebellar connection was right MDm - left Lob I–III (*d* = 0.81, large), while most others showed decreases (e.g., left CM - right Lob IV, *d* = −1.08, large negative). Figure 8 provides a complete overview of effect sizes for all connections involving medial thalamic nuclei, with colour gradients indicating task-related increases (red) and decreases (blue) in connectivity compared to baseline.

#### Lateral thalamic nuclei

Consistent with the anterior and medial thalamic groups, the lateral thalamic nuclei (VA, VLa, VLp, VPL) showed strong task-related increases in connectivity relative to baseline with sensorimotor regions during the tactile task. This pattern was broadly distributed across connections, particularly toward the left sensorimotor cortex ROIs. For example, large effects were observed for right VLa – left PoCG (*d* = 2.14), right VLp – left PoCG (*d* = 1.94), and left VLp – left PCG (*d* = 2.10), among many other sensorimotor connections showing similarly elevated values. In contrast, cerebellar connectivity during tactile stimulation was predominantly reduced, especially for connections from left thalamic nuclei, such as left VLp – left Lob VI (*d* = −1.90) and left VLa – left Lob VI (*d* = −1.72). During the motor task, the overall pattern was more variable. Increases in connectivity with sensorimotor cortex regions were still present (e.g., left VLa – left PoCG, *d* = 1.48), but other connections showed reductions (e.g., left VPL – left PCG, *d* = −1.62). In contrast to the tactile task, cerebellar connectivity was more strongly positive, with most connections showing small to moderate increases (e.g., right VLp – right Lob VI, *d* = 0.70), and relatively few small negative effects. These findings, visualized in Figure 9, show a shift in connectivity profiles across tasks: tactile stimulation was associated with widespread increases in sensorimotor connectivity, while the motor task led to stronger cerebellar engagement.

#### Posterior thalamic nuclei

Posterior thalamic nuclei (MGN, LGN, PuA, PuL, PuM, PuI) exhibited task-dependent changes in functional connectivity, quantified using Cohen’s *d* effect sizes calculated between each task condition and its corresponding baseline. Distinct patterns were observed for the motor and tactile tasks, as shown in Figure 10. This pattern closely resembled that of the lateral thalamic group, particularly in the task-specific engagement of sensorimotor and cerebellar regions. During the tactile task, posterior nuclei showed robust increases in connectivity with sensorimotor areas, especially in the left hemisphere. Notable examples included right PuL – left PoCG (*d* = 2.29, large), right PuL – left PrCG (*d* = 2.05), and left PuL – left PoCG (*d* = 1.82). These effects were distributed across both hemispheres, indicating a bilateral enhancement of sensorimotor connectivity during tactile stimulation. In contrast, cerebellar connectivity showed a more mixed pattern, with a combination of weak increases and moderate reductions (e.g., left PuI – left Lob VI, *d* = −1.10). In the motor task, the posterior group exhibited a shift toward stronger cerebellar connectivity, while sensorimotor effects were more variable. Large increases were observed for right PuI – right Lob V (*d* = 1.14) and right PuM – right Lob VI (*d* = 1.10). Some sensorimotor regions connections remained strongly positive (e.g., right PuM – left PrCG, *d* = 1.89), while others showed moderate decreases (e.g., left PuM – right PCG, *d* = −0.73).

## Discussion

The aim of this study was to extend our previous work (Charyasz et al., 2023) by conducting a detailed examination of task-dependent functional connectivity between thalamic nuclei and various cortical, subcortical, and cerebellar regions. While the results of the voxel-based analysis in our previous study identified the specific thalamic nuclei activated during both tasks, they did not provide any insight into the connections between these nuclei and different brain regions. To address this limitation, we analyzed our previously published dataset of task-based fMRI measurements at 9.4 T with a focus on the assessment of connectivity patterns during both an active motor (finger-tapping) task and a passive (tactile-finger) sensory task.

### Task-dependent and region-specific functional connectivity

The identification of both task-dependent as well as region-specific variability in the functional connectivity was an important part of our study. Specifically, the tactile task exhibited a higher number of significant connections compared to the motor task. The stronger connectivity of the thalamic nuclei with regions such as insula, IPL and PCAL suggests that sensory integration and attentional processing are enhanced during passive stimulation. These results are coherent with previous work suggesting that the thalamus does not simply act as a passive relay station but plays an active role in adapting sensory processing (Sherman, 2016; Hwang et al., 2017). Furthermore, the involvement of visual regions, including the cuneus and lingual gyrus, in the tactile task highlights the multisensory nature of thalamic processing (Barczak et al., 2023; Driver and Noesselt, 2008). In contrast, consistent with the role of the thalamus in motor coordination and proprioception (Bosch-Bouju et al., 2013; Semrau et al., 2015), the motor task showed more focused connectivity with cerebellar and cortical motor areas. The observed differences in strength and connectivity patterns between the two tasks show the ability of the thalamus to dynamically respond to the specific functional demands of different sensorimotor tasks. A more detailed discussion of how the nature of the task and inclusion of thalamic groups contribute to these connectivity patterns is provided below.

### Anterior thalamic nuclei (ATN)

The anterior thalamic nuclei - AV, LD and LP - are known for their role in memory, learning and attention. These nuclei are essential components of the Papez circuit (Papez, 1995), which connects the thalamus to the hippocampus and cingulate cortex to support cognitive and emotional functions (Jankowski et al., 2013; Grodd et al., 2020; Aggleton et al., 2010; Nelson, 2021). In addition to these functions, the ATN has also been implicated in integration, particularly in tasks requiring focused attention and sensory discrimination (Wright et al., 2015; Sweeney-Reed et al., 2017). Enhanced connectivity between the ATN and subcortical regions, including the cerebellar lobules and the basal ganglia (caudate, putamen, pallidum), was observed in both motor and tactile tasks, however with a higher number of pairwise connections in the tactile task. The direct involvement of the ATN in motor processes is not well-studied, but the connectivity observed here suggests a possible modulatory role in thalamo-striatal and thalamo-cerebellar pathways by supporting attention-driven sensorimotor integration.

The connectivity patterns specific to the two tasks show the versatility of the ATN. The motor task elicited increased connectivity of the ATN with the insula and IPL, which are associated with sensorimotor integration and movement planning (Chang et al., 2013; Mehler and Reschechtko, 2018). In contrast, the tactile task engaged a broader network, including the insula, the IPL, and the PCAL. This pattern of connectivity indicates a role for the ATN in multisensory integration and spatial representation (Jankowski et al., 2013). Furthermore, the observed reduction in connectivity with visual regions, such as the cuneus, can be interpreted as a process of neural reorganization that prioritizes tactile processing over visual input. This is consistent with the concept of flexible modulation within thalamocortical networks, which allows for task-specific reorganization depending on sensory demands (Sasaki et al., 2022).

### Medial thalamic nuclei

The medial thalamic nuclei (CM, Pf, MDm and MDl) are thought to act as important hubs in the thalamo-cortical and thalamo-striatal networks, which are involved in sensorimotor coordination, attention and higher-order cognitive functions (Jones, 2007; Kumar et al., 2023; Van der Werf et al., 2002). The tactile task demonstrates connectivity among all four medial thalamic nuclei, which is consistent with another study reporting that tactile processing involves a broader network of thalamic structures, demonstrating the ability of the thalamus to integrate multimodal sensory inputs (Habig et al., 2023; Wahlbom et al., 2021). The extensive involvement of thalamic nuclei in tactile tasks may enhance the complex sensory discrimination necessary for tactile signal processing exploration. The observed increase in connectivity between the medial thalamic nuclei and the basal ganglia (putamen, pallidum, and caudate) in both tasks supports previous findings that thalamo-striatal pathways play a role in motor and sensory signal processing (Smith et al., 2009). The consistent connectivity noted in both tasks shows that the thalamo-striatal network is not limited to specific tasks but may instead serve as an essential network for sensory integration.

Task-specific connectivity between medial thalamic nuclei and cortical regions, particularly the posterior cortical regions (cuneus, lingual gyrus, and PCAL), and the sensorimotor cortex (PCG, PoCG, and PrCG), also exhibit differences. While no connectivity was detected between the CM and Pf nuclei and the cuneus or lingual areas during the motor task, robust connectivity was evident during the tactile task. This finding is in line with those of previous studies showing that the lingual and the cuneus regions are involved in sensory processing and visual–spatial attention (Lee et al., 2020; Palejwala et al., 2021). An increased number of connections were found between sensorimotor cortex regions and the medial thalamic nuclei during the motor task, which aligns with the role of these nuclei in sensorimotor coordination, learning and decision making (Ilyas et al., 2019; Mitchell, 2015; Saalmann, 2014).

### Lateral thalamic nuclei

The lateral thalamic nuclei - VA, VL (VLa, VLp) and VPL - are referred to as the motor thalamic nuclei (Ilinsky et al., 2018) and play a crucial role in sensorimotor processing by acting as a hub for integrating and relaying sensory and motor information to various cortical and subcortical regions (Bosch-Bouju et al., 2013; Jones, 2007).

The enhanced connectivity between the lateral thalamic nuclei and cerebellar regions emphasizes the importance of the cerebello-thalamic pathway in motor coordination and sensory integration (Palesi et al., 2015; Pisano et al., 2021). In the motor task, increased connectivity was observed between the VLp, VPL, and cerebellar lobules (I-III, IV, V), aligning with the cerebellum’s role in delivering motor feedback and error correction during movement (Stoodley et al., 2012; Popa et al., 2016). Cerebellar outputs project to the VA and VL nuclei to augment motor execution, further supporting the role of the thalamus as a dynamic relay and control hub in motor signal processing (Gornati et al., 2018; Koster and Sherman, 2024). In the tactile task, increased connectivity was observed in the cerebellar lobules IV-VI with stronger involvement of the VL nuclei, indicating an involvement for these thalamic nuclei in the integration of tactile sensory input with motor adjustments.

Connectivity between the lateral thalamic nuclei and the basal ganglia (putamen, caudate, and pallidum) confirms their importance in human motor and sensory circuits. The VA, VL and VPL nuclei show increased connectivity with the putamen and pallidum in the motor task, suggesting their involvement in the cortico-basal ganglia-thalamo-cortical loop. Evidence from human studies corroborates this circuit, demonstrating that basal ganglia outputs modulate thalamic activity to ensure motor execution (Draganski et al., 2008; Mohagheghi Nejad et al., 2018; McFarland and Haber, 2002). Enhanced connectivity with subcortical regions in the tactile task indicates a greater demand for sensory integration and processing, consistent with the concept of task-specific recruitment of neural resources (Jang et al., 2017; Sasaki et al., 2022).

The lateral thalamic nuclei exhibit a higher number of significant connections with sensorimotor cortical regions during the motor task compared to the tactile task. This variability suggests that the thalamic nuclei may have distinct roles in modulating or integrating signals based on the complexity and demands of tasks (Wang, 2024; Halassa and Kastner, 2017). The PrCG consistently showed connection with the VA, VLa, VLp, and VPL nuclei across tasks, supporting the integration of motor-related signals with sensory feedback for movement planning and execution. This is in line with research that shows how the VL and VPL nuclei relay information from the cerebellum and basal ganglia to cortical motor regions, ensuring coordinated and precise movement (Middleton and Strick, 2000; Sommer, 2003). The PoCG exhibited consistent connection with the bilateral VPL nuclei in both tasks, emphasizing the VPL’s role in relaying tactile information. This finding aligns with research demonstrating that the VPL supports sensory integration by relaying inputs from ascending pathways to cortical regions (Studtmann et al., 2023). Decreased connectivity between the VA nucleus and bilateral cuneus, as well as the bilateral SPL may be provoked by the active suppression of task-irrelevant regions (Tomasi et al., 2014).

### Posterior thalamic nuclei

The posterior thalamic nuclei - LGN, MGN, PuA, and PuM – play a crucial role in sensory and cognitive processing by integrating and transmitting visual, auditory, and multisensory information, as well as supporting attention, sensory prioritization, and cognitive control (Saalmann et al., 2012; Saalmann and Kastner, 2011; Nagalski et al., 2016; Meng and Schneider, 2022). These nuclei show distinct patterns of connectivity with cerebellar, subcortical, and cortical regions, reflecting their adaptive roles in sensory and motor processing. In the motor task, increased connectivity was observed in the LGN, MGN, PuA, and PuM and cerebellar lobules I-III, IV, and V. The LGN’s involvement is consistent with its role in relaying visual information essential for visuomotor coordination (Casagrande et al., 2005; Lesica and Stanley, 2005). Similarly, the MGN’s connections might reflect the importance of auditory-motor integration for tasks requiring timing and rhythm (O'Connor et al., 1997). The pulvinar nuclei (PuA and PuM) exhibit functional connectivity with these cerebellar lobules, supporting visuomotor coordination and attentional control, aligning with their known role in modulating sensorimotor processing (Bridge et al., 2016; Froesel et al., 2021; Saalmann and Kastner, 2011).

The tactile task elicits connectivity with cerebellar lobules IV-VI, with the LGN nuclei exhibiting stronger interactions, suggesting their involvement in tactile sensory processing and attentional modulation. Thalamic connectivity with basal ganglia regions, including the putamen, pallidum, and caudate, remains consistent across tasks. In both tasks, all posterior group nuclei exhibit strong connectivity with basal ganglia regions, suggesting their involvement in voluntary movements and visual signal processing (Wilke et al., 2018; Cortes et al., 2024; Shimono et al., 2012).

Thalamocortical connectivity patterns also differed between tasks. In the motor task, the LGN, MGN, and pulvinar nuclei show connections with sensorimotor regions, including the PrCG, PoCG, PCG and SPL. These connections highlight their role in motor execution, sensory feedback integration, and visuospatial processing, consistent with research on the role of the thalamus in coordinating sensorimotor pathways (Basile et al., 2024). In the tactile task, the pulvinar nuclei exhibit enhanced connectivity with the insula and IPL, which are regions associated with tactile information processing and attentional regulation (Barron et al., 2015; Homman-Ludiye and Bourne, 2019).

### Summary of findings

Our results reveal distinct task-specific functional connectivity patterns in the anterior, medial, lateral, and posterior thalamic nuclei. Instead of acting as static hubs with fixed roles in neural signal processing, these nuclei function as versatile hubs that dynamically adjust their functional connections in response to the demands of different tasks. During the motor task, thalamic networks were primarily involved in motor planning, execution, and proprioceptive feedback, whereas the tactile task elicited broader connectivity with regions associated with sensory integration, attentional control, and visual processing. This confirms and extends previous observations of the heterogeneity of thalamic nuclei in sensorimotor processing (Kumar et al., 2022; Zhou et al., 2016; Saalmann and Kastner, 2015; Acsády, 2023).

### Exploratory findings: task condition vs. baseline condition

As described in the Methods section, exploratory analyses were conducted using an uncorrected threshold of *p* < 0.01 to examine condition-related differences in functional connectivity that may not have been detectable due to limited statistical power. A contrast analysis comparing the motor condition against the baseline (finger tapping > baseline) revealed both increases and decreases in functional connectivity between thalamic nuclei and cortical, subcortical, and cerebellar regions (Supplementary Figure S3). Increased connectivity was observed between several posterior thalamic nuclei – including the left LGN, bilateral PuM, bilateral PuI – and the right cerebellar lobule V, as well as between the right PuM and the right cerebellar lobule VI. Connectivity was also increased between the left VLa and the left PrCG, consistent with the engagement of motor-related circuits during task execution and suggesting enhanced thalamocerebellar and thalamocortical coupling during movement execution. In contrast, decreased connectivity was found between several thalamic nuclei (AV, LP, VA, VPL, MDm, MGN) and a range of cortical and subcortical regions, including the PoCG, IPL, insula, pallidum, caudate, and putamen.

Exploratory analysis of the tactile > baseline contrast revealed a distinct set of functional connections that were enhanced during the tactile. These findings included increased connectivity between several thalamic nuclei (e.g., AV, LP, VLa, VLp, VPL, MDl, PuM, PuA, PuI) and various sensorimotor regions - such as the PoCG, PCG, PRG, and the left insula—although not all nuclei were connected to all regions. These patterns are presented in detail in Supplementary Figure S4.

Both tasks were contrasted against the same type of fixation baseline, which required continuous visual attention but no active sensorimotor engagement. Despite this common baseline, the tactile condition elicited broader changes in connectivity, likely due to its combined sensory and cognitive demands: participants were required not only to perceive rhythmic tactile stimulation but also to count missing pulses, engaging both thalamocortical sensory circuits and higher-order attentional systems. In comparison, the motor task involved repetitive, visually guided finger movements that may have relied on more automatized motor circuits.

These findings align with prior work showing that fixation or rest conditions are not truly inactive (Stark and Squire, 2001). They are also consistent with the view that task-evoked functional connectivity reflects modulations of a stable intrinsic network architecture, with limited reconfiguration depending on task complexity and demands (Cole et al., 2014). However, given the exploratory nature of these results and the small sample size, further studies with larger cohorts are required to validate these findings and to clarify the task- and baseline-specific dynamics of thalamic connectivity.

### Limitations and future directions

Although this study provides valuable insights, certain limitations must be acknowledged. The study relies on the relatively small sample size, the use of stimuli applied only to the right hand, and the challenges related to accurate segmentation of thalamic nuclei due to altered tissue contrast at 9.4 T as compared to lower clinical field strengths. Nevertheless, the dataset provided sufficient spatial resolution and signal quality to investigate task-dependent activation and connectivity patterns and provide robust insights into the functional role of the thalamic nuclei. The understanding of thalamic connectivity could be further improved by addressing these limitations in future research. Increasing the sample size, applying stimulation to both hands and using improved segmentation techniques may provide a more comprehensive view of the thalamic function. An extended range of performed sensorimotor tasks would also help to confirm and extend these findings. Furthermore, investigating thalamic connectivity in clinical populations, such as Parkinson’s disease patients (Halliday, 2009; Wang et al., 2021), could provide important information on how connectivity alterations affect sensorimotor impairments.

To the best of our knowledge, this study is the first to show comprehensive results of task-dependent functional connectivity between thalamic nuclei and cortical, subcortical, and cerebellar regions using task-based fMRI at a field strength of 9.4 T, which indicates the role of the thalamus as a flexible hub for sensorimotor integration. This study extends our current understanding of thalamic functional heterogeneity by demonstrating motor and tactile connectivity patterns. Mapping these dynamic connectivity patterns has important implications for future research on thalamic function in healthy and clinical populations.

## Data Availability

The raw data supporting the conclusions of this article will be made available by the authors, without undue reservation.

## Footnotes

^1^ http://www.fil.ion.ucl.ac.uk/spm

^2^ http://surfer.nmr.mgh.harvard.edu

## Supplementary material

Supplementary Figure S1 Subject-level ROI-to-ROI connectivity matrices for the motor and tactile tasks (Subjects 1–4). Each subject is represented by a pair of connectivity matrices, with the motor condition on the left and the tactile condition on the right. The colour scale represents Fisher Z-transformed correlation values, ranging from negative (blue) to positive (red), indicating the strength of functional connectivity. ROIs (1–66) are ordered identically to those in Figures 1, 6. ROI order: Cortical regions (L-PCG, L-PoCG, L-PrCG, R-PCG, R-PoCG, R-PrCG, L-SPL, L-IPL, L-Cun, L-PCAL, L-LING, L-INS, R-SPL, R-IPL, R-Cun, R-PCAL, R-LING, R-INS), Basal ganglia regions (L-Cd, L-Pu, L-Pd, R-Cd, R-Pu, R-Pd), Thalamic nuclei (L-AV, L-LD, L-LP, R-AV, R-LD, R-LP, L-VA, L-VLa, L-VLp, L-VPL, R-VA, R-VLa, R-VLp, R-VPL, L-CM, L-Pf, L-MDm, L-MDl, R-CM, R-Pf, R-MDm, R-MDl, L-MGN, L-LGN, L-PuA, L-PuL, L-PuM, L-PuI, R-MGN, R-LGN, R-PuA, R-PuL, R-PuM, R-PuI), Cerebellar lobules (L-Lob I-III, L-Lob IV, L-Lob V, L-Lob VI, R-Lob I-III, R-Lob IV, R-Lob V, R-Lob VI).

Supplementary Figure S2 Subject-level ROI-to-ROI connectivity matrices for the motor and tactile tasks (Subjects 5–8). Each subject is represented by a pair of connectivity matrices, with the motor condition on the left and the tactile condition on the right. The colour scale represents Fisher Z-transformed correlation values, ranging from negative (blue) to positive (red), indicating the strength of functional connectivity. ROIs (1–66) are ordered identically to those in Figures 1, 6, as well as Supplementary Figure S1.

Supplementary Figure S3 ROI-to-ROI functional connectivity matrix for the Motor condition > Baseline condition contrast, computed using GLM contrast weights [1 −1] and thresholded at *p* < 0.01 (uncorrected, connection level). From the total of 66 ROIs, only 34 ROIs are shown, representing those for which at least one statistically significant connection was identified. ROIs are ordered and labelled according to anatomical areas (cortical, subcortical, thalamic, cerebellar), with a consistent arrangement along both axes. The colour scale represents T-values, ranging from negative (blue) to positive (red).

Supplementary Figure S4 ROI-to-ROI functional connectivity matrix for the Tactile condition > Baseline condition contrast, computed using GLM contrast weights [1 −1] and thresholded at *p* < 0.01 (uncorrected, connection level). From the total of 66 ROIs, only 41 ROIs are shown, representing those for which at least one statistically significant connection was identified. ROIs are ordered and labelled according to anatomical areas (cortical, subcortical, thalamic, cerebellar), with a consistent arrangement along both axes. The colour scale represents T-values, ranging from negative (blue) to positive (red).

## Glossary

AC: anterior commissure
AV: anteroventral
ATN: anterior thalamic nuclei
BOLD: blood-oxygen-level-dependent
CM: centromedian
Cun: cuneus
EPI: echo planar imaging
FA: flip angle
fMRI: functional magnetic resonance imaging
FOV: field of view
FWHM: full width at half maximum
GLM: general linear model
HRF: hemodynamic response function
INS: insula
IPL: inferior parietal lobule
LD: lateral dorsal
LGN: lateral geniculate
LING: lingual gyrus
LP: lateral posterior
MDl: lateral subdivision of mediodorsal thalamus
MDm: medial subdivision of mediodorsal thalamus
MGN: medial geniculate
MPRAGE: magnetization-prepared rapid acquisition gradient echo
PCG: paracentral gyrus
PCAL: pericalcarine
PC: posterior commissure
Pf: parafascicular
PoCG: postcentral gyrus
PuA: anterior pulvinar
PuI: inferior pulvinar
PuL: later pulvinar
PuM: medial pulvinar
PrCG: precentral gyrus
GRAPPA: acceleration factor
SPL: superior parietal lobule
TE: echo time
TR: repetition time
VA: ventral anterior
VL: ventral lateral
VLa: ventral lateral anterior
VLp: ventral lateral posterior
VP: ventral posterior
VPL: ventral posterior lateral
