# Supplementary figures and images for: Functional connectivity of thalamic nuclei during sensorimotor task-based fMRI at 9.4 Tesla
Source: Front Neurosci. 2025 May 13;19:1568222. doi: 10.3389/fnins.2025.1568222 (PMC12106322; doi:10.3389/fnins.2025.1568222)

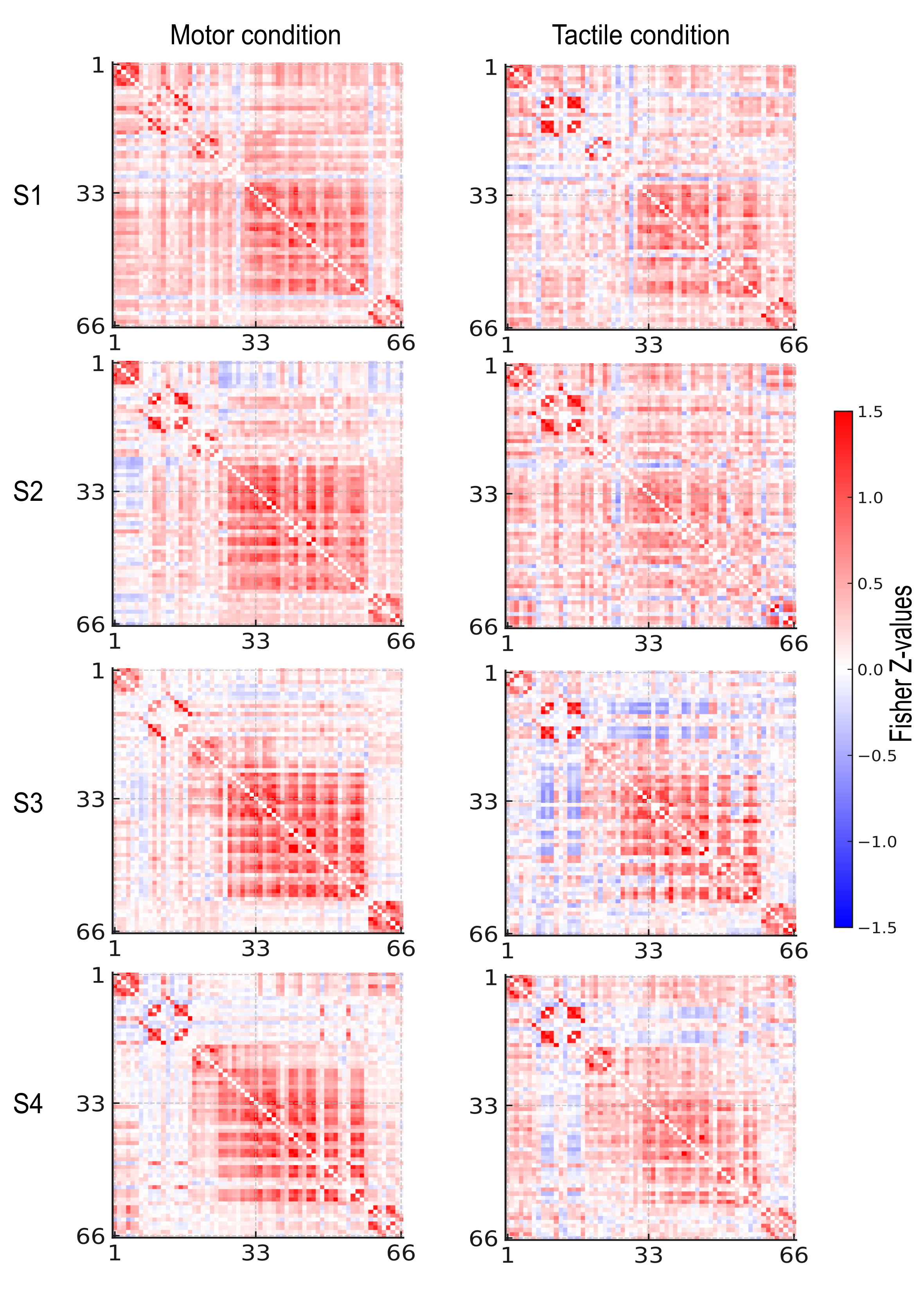

Supplement: Supplementary Figure S1 — Subject-level ROI-to-ROI connectivity matrices for the motor and tactile tasks (Subjects 1–4). Each subject is represented by a pair of connectivity matrices, with the motor condition on the left and the tactile condition on the right. The colour scale represents Fisher Z-transformed correlation values, ranging from negative (blue) to positive (red), indicating the strength of functional connectivity. ROIs (1–66) are ordered identically to those in Figures 1, 6. ROI order: Cortical regions (L-PCG, L-PoCG, L-PrCG, R-PCG, R-PoCG, R-PrCG, L-SPL, L-IPL, L-Cun, L-PCAL, L-LING, L-INS, R-SPL, R-IPL, R-Cun, R-PCAL, R-LING, R-INS), Basal ganglia regions (L-Cd, L-Pu, L-Pd, R-Cd, R-Pu, R-Pd), Thalamic nuclei (L-AV, L-LD, L-LP, R-AV, R-LD, R-LP, L-VA, L-VLa, L-VLp, L-VPL, R-VA, R-VLa, R-VLp, R-VPL, L-CM, L-Pf, L-MDm, L-MDl, R-CM, R-Pf, R-MDm, R-MDl, L-MGN, L-LGN, L-PuA, L-PuL, L-PuM, L-PuI, R-MGN, R-LGN, R-PuA, R-PuL, R-PuM, R-PuI), Cerebellar lobules (L-Lob I-III, L-Lob IV, L-Lob V, L-Lob VI, R-Lob I-III, R-Lob IV, R-Lob V, R-Lob VI). [file Image_1.tiff]

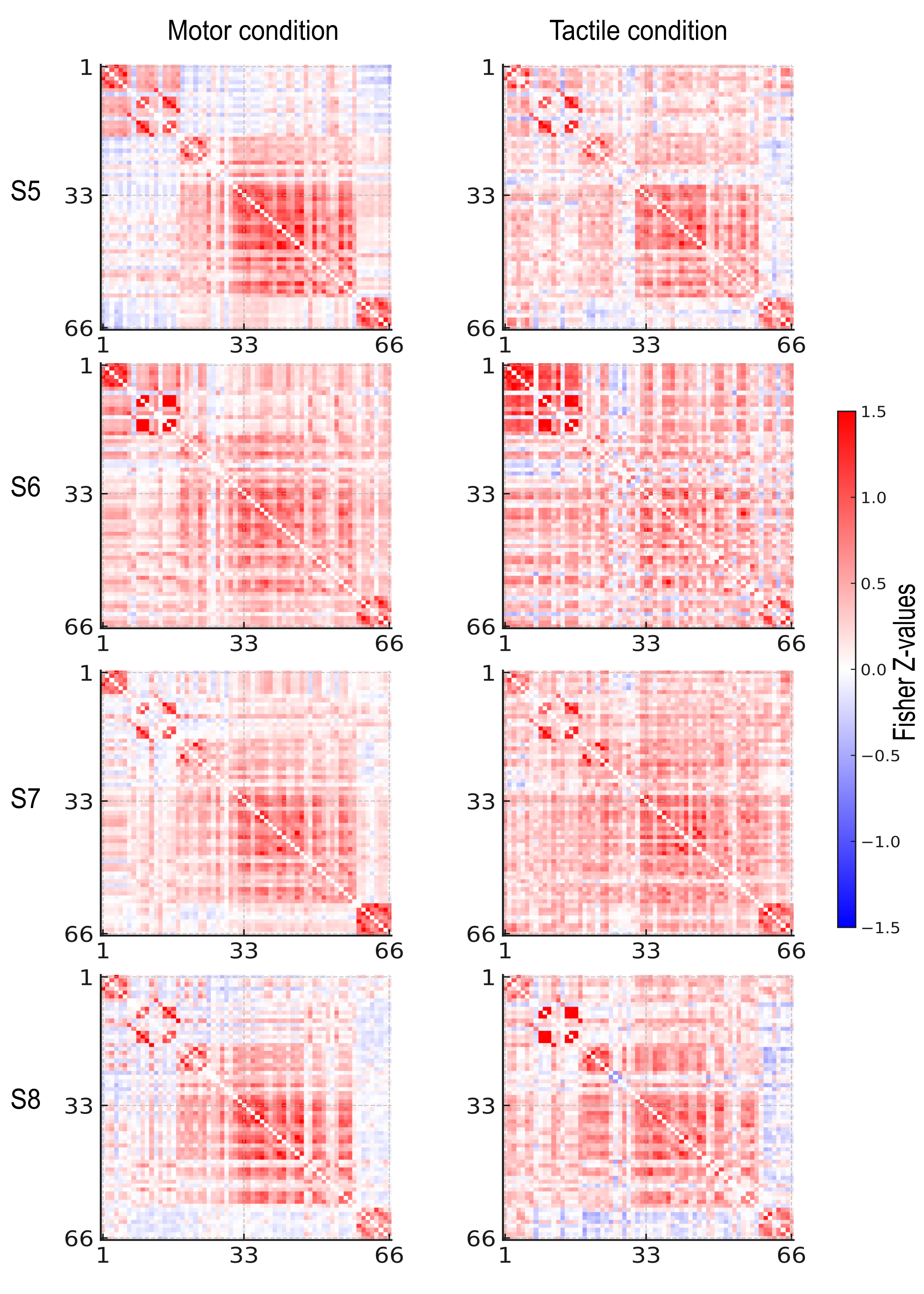

Supplement: Supplementary Figure S2 — Subject-level ROI-to-ROI connectivity matrices for the motor and tactile tasks (Subjects 5–8). Each subject is represented by a pair of connectivity matrices, with the motor condition on the left and the tactile condition on the right. The colour scale represents Fisher Z-transformed correlation values, ranging from negative (blue) to positive (red), indicating the strength of functional connectivity. ROIs (1–66) are ordered identically to those in Figures 1, 6, as well as Supplementary Figure S1. [file Image_2.tiff]

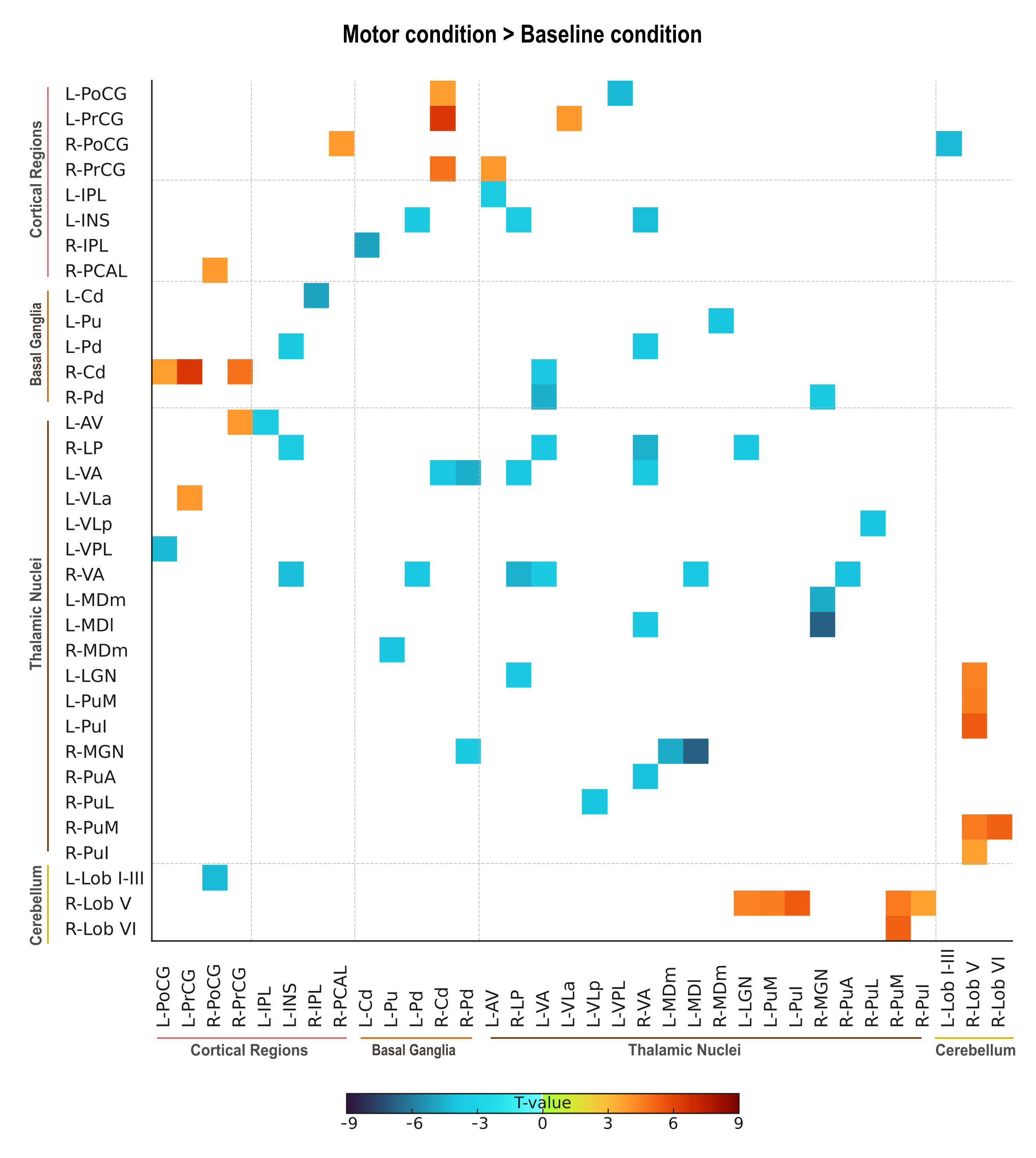

Supplement: Supplementary Figure S3 — ROI-to-ROI functional connectivity matrix for the Motor condition > Baseline condition contrast, computed using GLM contrast weights [1 −1] and thresholded at p < 0.01 (uncorrected, connection level). From the total of 66 ROIs, only 34 ROIs are shown, representing those for which at least one statistically significant connection was identified. ROIs are ordered and labelled according to anatomical areas (cortical, subcortical, thalamic, cerebellar), with a consistent arrangement along both axes. The colour scale represents T-values, ranging from negative (blue) to positive (red). [file Image_3.tiff]

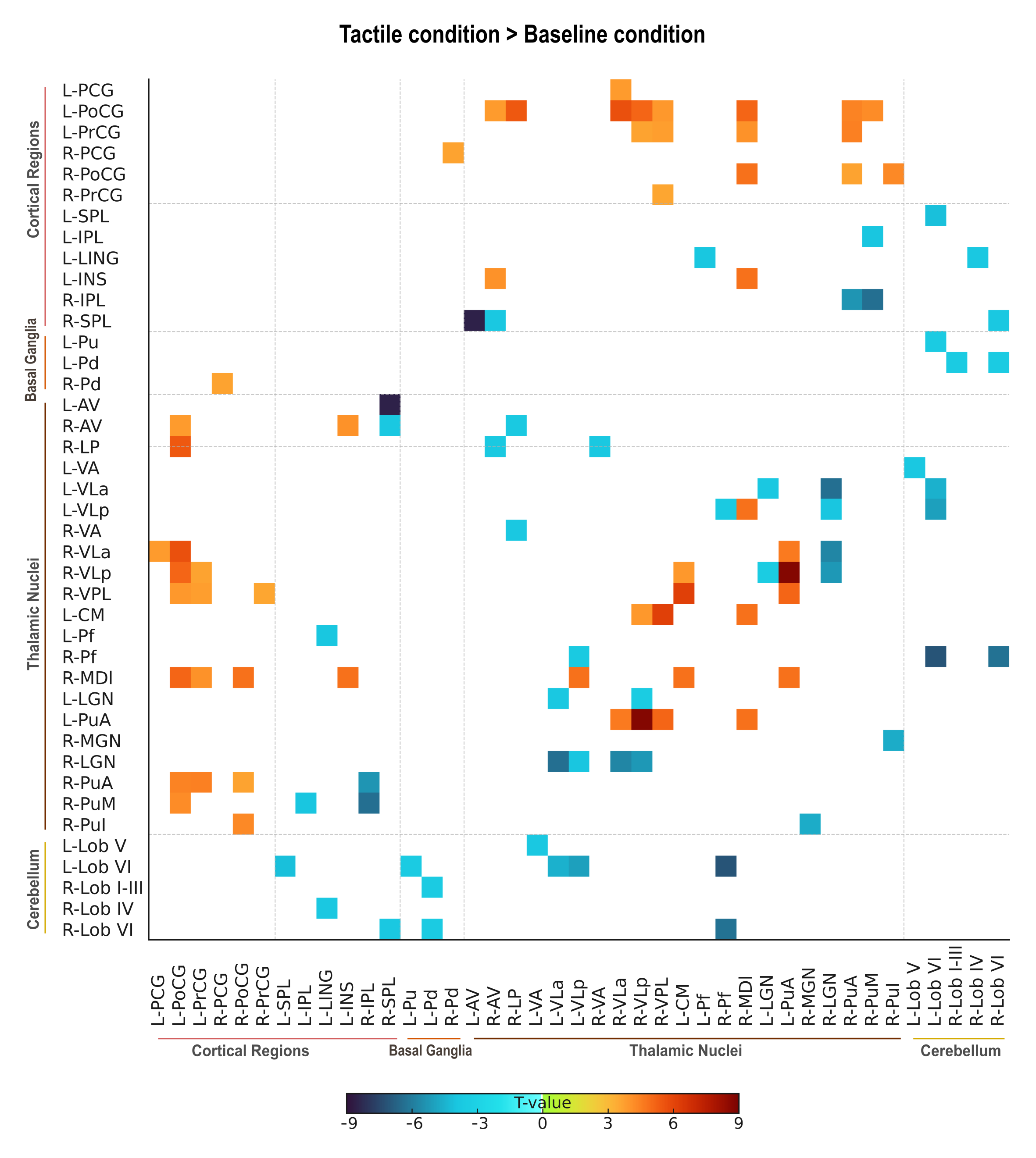

Supplement: Supplementary Figure S4 — ROI-to-ROI functional connectivity matrix for the Tactile condition > Baseline condition contrast, computed using GLM contrast weights [1 −1] and thresholded at p < 0.01 (uncorrected, connection level). From the total of 66 ROIs, only 41 ROIs are shown, representing those for which at least one statistically significant connection was identified. ROIs are ordered and labelled according to anatomical areas (cortical, subcortical, thalamic, cerebellar), with a consistent arrangement along both axes. The colour scale represents T-values, ranging from negative (blue) to positive (red). [file Image_4.tiff]
